# The epidemiology and estimated etiology of pathogens detected from the upper respiratory tract of adults with severe acute respiratory infections in multiple countries, 2014–2015

**DOI:** 10.1371/journal.pone.0240309

**Published:** 2020-10-19

**Authors:** Jennifer Milucky, Tracy Pondo, Christopher J. Gregory, Danielle Iuliano, Sandra S. Chaves, John McCracken, Adel Mansour, Yuzhi Zhang, Mohammad Abdul Aleem, Bernard Wolff, Brett Whitaker, Toni Whistler, Clayton Onyango, Maria Renee Lopez, Na Liu, Mohammed Ziaur Rahman, Nong Shang, Jonas Winchell, Malinee Chittaganpitch, Barry Fields, Herberth Maldonado, Zhiping Xie, Stephen Lindstrom, Katherine Sturm-Ramirez, Joel Montgomery, Kai-Hui Wu, Chris A. Van Beneden, the Adult TAC Working Group

**Affiliations:** 1 Division of Bacterial Diseases, Centers for Disease Control and Prevention, National Center for Immunization and Respiratory Diseases, Atlanta, Georgia, United States of America; 2 Division of Global Health Protection, Centers for Disease Control and Prevention, Thailand Ministry of Public Health, Thailand; 3 Influenza Division, Centers for Disease Control and Prevention, National Center for Immunization and Respiratory Diseases, Atlanta, Georgia, United States of America; 4 Influenza Division, Centers for Disease Control and Prevention, National Center for Immunization and Respiratory Diseases, CDC Kenya Office, Kenya; 5 Center for Health Studies, Universidad del Valle de Guatemala, Guatemala City, Guatemala; 6 Division of Global Health Protection, Centers for Disease Control and Prevention, Egypt; 7 Division of Global Health Protection, Centers for Disease Control and Prevention, China; 8 International Centre for Diarrheal Disease Research, Dhaka, Bangladesh; 9 Division of Viral Diseases, Centers for Disease Control and Prevention, National Center for Immunization and Respiratory Diseases, Atlanta, Georgia, United States of America; 10 Kenya Medical Research Institute/Centers for Disease Control and Prevention Public Health Collaboration, Kisumu, Kenya; 11 China Centers for Disease Control and Prevention, National Institute for Viral Disease, Beijing, China; 12 Thailand Ministry of Public Health, National Institute of Health, Bangkok, Thailand; 13 Division of Global Health Protection, Centers for Disease Control and Prevention, Kenya; 14 Influenza Division, Centers for Disease Control and Prevention, National Center for Immunization and Respiratory Diseases, CDC Bangladesh Office, Bangladesh; 15 Division of Global Health Protection, Centers for Disease Control and Prevention, Atlanta, Georgia, United States of America; Ben-Gurion University of the Negev, UNITED STATES

## Abstract

**Introduction:**

Etiology studies of severe acute respiratory infections (SARI) in adults are limited. We studied potential etiologies of SARI among adults in six countries using multi-pathogen diagnostics.

**Methods:**

We enrolled both adults with SARI (acute respiratory illness onset with fever and cough requiring hospitalization) and asymptomatic adults (adults hospitalized with non-infectious illnesses, non-household members accompanying SARI patients, adults enrolled from outpatient departments, and community members) in each country. Demographics, clinical data, and nasopharyngeal and oropharyngeal specimens were collected from both SARI patients and asymptomatic adults. Specimens were tested for presence of 29 pathogens utilizing the Taqman^®^ Array Card platform. We applied a non-parametric Bayesian regression extension of a partially latent class model approach to estimate proportions of SARI caused by specific pathogens.

**Results:**

We enrolled 2,388 SARI patients and 1,135 asymptomatic adults from October 2013 through October 2015. We detected ≥1 pathogen in 76% of SARI patients and 67% of asymptomatic adults. *Haemophilus influenzae* and *Streptococcus pneumoniae* were most commonly detected (≥23% of SARI patients and asymptomatic adults). Through modeling, etiology was attributed to a pathogen in most SARI patients (range among countries: 57.3–93.2%); pathogens commonly attributed to SARI etiology included influenza A (14.4–54.4%), influenza B (1.9–19.1%), rhino/enterovirus (1.8–42.6%), and RSV (3.6–14.6%).

**Conclusions:**

Use of multi-pathogen diagnostics and modeling enabled attribution of etiology in most adult SARI patients, despite frequent detection of multiple pathogens in the upper respiratory tract. Seasonal flu vaccination and development of RSV vaccine would likely reduce the burden of SARI in these populations.

## Introduction

Lower respiratory tract infections (LRTI) are a leading cause of morbidity and mortality worldwide, accounting for 4.4% of deaths among all ages [1]. Globally, decreases in mortality caused by LRTIs have been observed following the introduction of *Haemophilus influenzae* type b, pertussis, and pneumococcal conjugate vaccines. However, LRTI remain a leading cause of mortality among young children and elderly in low- and middle-income countries [2].

Few studies have systematically identified the contributions of both bacteria and viruses to acute respiratory tract infections in adults. Most have been conducted among pneumonia patients in high-income countries [3–8], among children [9], or have used limited diagnostic testing [10]; only one meta-analysis has looked at viral etiologies of acute respiratory tract infections in adults across multiple countries [11]. Studies in resource-rich countries may not be applicable to low- and middle-income countries due to differences in pathogen prevalence and variations in risk factors for disease (e.g., hygiene, access to clean water, malnutrition, crowding, HIV prevalence, health care access, vaccination rates, air pollution) among the populations.

Because a variety of pathogens that cause acute respiratory infections produce similar clinical presentations, etiology studies require laboratory diagnostics covering a wide range of pathogens. The Taqman^®^ Array Card (TAC, Thermo Fisher Scientific, Carlsbad, CA) is a microfluidic, multiple pathogen detection tool that uses solid-phase real-time PCR technology for which analytical and laboratory validation on clinical specimens has been performed [12]. In conjunction with other diagnostic tests, the TAC platform has been used to test a variety of specimen types in large etiology studies of neonatal infection in Africa and South Asia [13, 14], but its application to respiratory disease surveillance has been limited to a few studies in the United States [8, 15, 16].

We used the TAC platform in diverse global sites to better understand the potential contributions of viral and bacterial pathogens in severe acute respiratory infections (SARI) among adults in middle-income countries. We describe here the pathogens detected in upper respiratory tract samples collected from both adults with SARI and adults without respiratory symptoms. We also estimate of the etiologic fraction of SARI attributed to individual pathogens.

## Methods

In collaboration with the Centers for Disease Control and Prevention’s country offices, we conducted a multi-site study of the etiologies of SARI among hospitalized adults in six lower middle income or upper middle income countries during 2013–2015 [17]. We also enrolled healthy adults without symptoms of respiratory infection to estimate background carriage rates of TAC pathogens from the same communities. In each site we conducted the study for ≥12 months. The local institutional review board of each country reviewed and approved the study [CDC's Human Research Protection Office reviewed and approved the request to continue reliance on a non-CDC IRB for “Hospital Based Human Influenza Surveillance in Bangladesh (HBIS)” in accordance with 45 CFR 46.114. The protocol has been reviewed and approved by the ICDDR, B IRB. #6458; Approved by the Institute of Viral Disease Control and Prevention, CDC China-IRB00001071; Approved by US Navy Medical Research Unit No. 3 IRB project 906 (Egypt); Approved by Universidad del Valle de Guatemala IRB- protocol 072-10-2012; Approved by Kenya Medical Research Institute protocol 2558; Approved by Thailand MoPH Ethical Review Committee and Centers for Disease Control and Prevention; Protocol 6567]. Written consent was obtained from study participants. If a participant was incapacitated and unable to provide consent, a guardian provided consent following local IRB requirements.

### Characteristics of participating sites

In each country, one or more hospitals conducted ongoing population-based or sentinel surveillance for SARI. Site descriptions are found in Table 1.

**Table 1:** Characteristics of participating sites.

Country	Sources of asymptomatic adult participants^a^	Participating sites	Estimated catchment population of adults	Number of hospitals	Number (%) of hospitals with ICU and ventilator capacity, respectively	Method of estimating catchment population^b^	Vaccines available during enrollment period
Bangladesh	Inpatients admitted with non-infectious illness to medical wards in tertiary care hospitals	Mymensing, Rajshahi, Kishoregonj, Bogra, Dinajpur, Comilla, Chittagong, Khulna, Jessore, Sylhet, and Barisal	2,658,739	11	6 (55%); 5 (45%)	Hospital admissions data survey and catchment area hospital utilization survey (HUS)	Hib
(Lower middle-income country)							
China	Non-household members accompanying SARI patients	Chenzhou city	1,066,061	1	1 (100%), 1 (100%)	Hospital admissions data survey	PCV (not routinely used)
(Upper middle-income country)							
							Hib (not routinely used)
Egypt	Inpatient adults from OB/GYN, orthopedic and surgical units; outpatients from dental and family planning clinics	Damanhour city	122,679	3	3 (100%), 3 (100%)	HUS	Hib
(Lower middle-income country)							
Guatemala	Inpatients from orthopedic and trauma wards	Santa Rosa, Quetzaltenango, and Guatemala City	371,895	3	3 (100%), 3 (100%)	HUS and population projections	PCV13;
(Upper middle-income country)							Hib
Kenya	Community-based asymptomatic adults^c^	Lwak and Siaya county	22,361	2	1 (50%), 0	HUS	PCV10;
(Lower middle-income country)							Hib
Thailand	Outpatient departments	Nakhon Phanom province	86,916	4	1 (25%), 1 (25%)	HUS	PCV (not routinely used)
(Upper middle-income country)							
							Hib (Not routinely used)

^a^ Asymptomatic adult participants are adults without any of the following symptoms in the prior week: shortness of breath, fever, cough, runny nose, or sore throat.

^b^ Estimates of the catchment population were available to calculate incidence rates and confidence intervals for all SARI cases enrolled in Bangladesh, China, Egypt, Thailand, Guatemala (Quetzaltenango and Santa Rosa sites only), and Kenya (Karemo and the population-based surveillance system in Lwak only). Patients from sentinel surveillance sites without population-based surveillance but where denominators for participating sentinel hospitals could be estimated were included in incidence calculations.

^c^ The community-based asymptomatic adults were selected from a population in the community that undergo biweekly home visits to assess symptoms of illness. These households are visited biweekly and asked a series of questions about illness and healthcare exposures. Adults with no symptoms of infection reported at the time of visit (or in the prior 7 days) were asked to participate in this study.

### Case definition and enrollment

We enrolled adults (aged ≥18 years) with infections that met the WHO SARI case definition (acute respiratory infection with history of fever or measured fever of ≥ 38°C and cough, with onset within the last 7 days [Bangladesh, China, Egypt, and Thailand] or onset within the last 10 days [Guatemala and Kenya], and requiring hospitalization) because it is an established case definition used globally [18]. Patients were included if they lived in the defined catchment area and were enrolled into the study within 24 hours of hospital admission. Study staff identified eligible participants and collected required data from study participants during their hospital day and from medical records. To exclude nosocomial infections and convalescent SARI patients, we excluded patients who were hospitalized in the previous 14 days or who had an episode of clinician-diagnosed pneumonia or a previous enrollment into the study in the prior 30 days. For all sites except Guatemala, pneumonia was defined as consolidation, infiltrate, or pleural effusion noted on chest radiograph, as indicated in medical records. In Guatemala, radiologists reviewing chest x-rays defined pneumonia using WHO guidance [19].

### Enrollment of asymptomatic adults

To assess the association of a pathogen detected from an upper respiratory tract of a patient with SARI and not bacterial carriage or asymptomatic viral infection, we compared results of TAC testing among SARI patients to those from asymptomatic adults. Asymptomatic adults were included to estimate positivity for TAC pathogens among the population when there is no disease. We defined asymptomatic adults as adults without any of the following signs or symptoms in the prior week: shortness of breath, fever, cough, runny nose, or sore throat. We enrolled two asymptomatic adults for every five SARI patients, frequency matched by age group (18–49 years, 50–64 years, and ≥65 years), month of enrollment, catchment area, and HIV infection status (Kenya only) [20]. Sources of asymptomatic adult participants by site are detailed in Table 1. Asymptomatic adults were excluded from the study if they had been hospitalized in the past 14 days or had an episode of pneumonia in the previous 30 days.

### Specimen and data collection

Nasopharyngeal (NP) and oropharyngeal (OP) swabs were collected from each case within the first 24 hours of hospital admission. A case report form was completed to capture epidemiologic and clinical information. Discharge diagnoses were collected in all sites except Bangladesh. NP and OP specimens and a form capturing demographics, underlying health conditions, and antibiotic exposure were also collected from each asymptomatic adult.

### Sample size calculations

We based sample size calculations on an expected prevalence in the naso- or oropharynx of asymptomatic colonization with *Streptococcus pneumoniae* as detected by culture (approximately 20% colonization among asymptomatic adults in developed countries) [21, 22] or subclinical infection with rhinovirus (approximately 2.8% of asymptomatic adults from a study in Thailand) [23]. Each site received enough TACs to test approximately 700 specimens. To determine the best ratio (or distribution) of the number of tests to be used for TAC patients and for asymptomatic adults, we applied the concept of population attributable risk (PAR) to estimate the etiology fraction of pathogens detected [24]. Using the PROC POWER function of SAS software (version 9.4; SAS Institute, Cary, NC), we estimated that enrolling 500 case patients and 200 asymptomatic adults over a ≥12-month period at each site would provide a sample size sufficient to measure an etiologic fraction (PAR) of 12.9% or larger for pneumococcus and 5.4% or larger for rhinovirus, with a power of 80% and significance level of 0.05.

### Catchment population estimations and incidence calculations

To calculate the incidence of SARI and enable comparisons across sites and to historical data, estimates of the adult catchment population for each sentinel hospital were needed. The estimated catchment population and methods for obtaining this population by site are outlined in Table 1 [25–32].

### Laboratory methods

NP and OP specimens collected from each study participant were combined into one transport vial containing universal transport media. Total nucleic acid was extracted and tested using the TAC platform for the presence of 29 viral, bacterial and fungal pathogens. Additional details on laboratory methods and interpretation of results are found in laboratory supplemental materials (S1 File).

### Statistical analyses

We analyzed the frequency of basic demographics, underlying medical conditions, antibiotic use, and pathogens detected among SARI patients and asymptomatic adults. We calculated odds ratios to assess the association of each pathogen with SARI infection. We next assessed the attribution of etiology using a Bayesian modeling approach [33] to estimate the proportion of SARI cases attributed to each pathogen. According to this method, we assumed each SARI episode to have only one underlying pathogen cause. The relationship between the observed TAC results and pathogen etiology attribution was summarized by a linear mixture model of pathogen classes (detailed methods are included in S2 File). The complete pathogen class list included all pathogens on the TAC card plus one additional class (“other/none”) for SARI cases that could not be attributed to any of the TAC pathogens. This class included causative pathogens which were not on the TAC, pathogens which may be causative but not present in the NP/OP specimen, and non-infectious causes. Pathogen proportions—the estimated proportion of SARI episodes attributed to each pathogen tested for by TAC—could vary by study site, age at onset, and date of enrollment.

False positive rates (i.e. the proportion of positive test results for a pathogen among cases and asymptomatic adults that were not attributed to the pathogen) varied by covariate values, while true positive rates (i.e. the proportion of positive test results for a pathogen among cases attributed to that pathogen) were constant (see S2 File for more detail). Only the true positive rate was assumed to be the same across all sites.

Data from all sites were combined in the model; however, pathogens included in the model varied across the study sites and were pre-selected by a stepwise procedure (S2 File) before building the linear-mixture model. For pathogens included in some sites but not others, the pathogen proportion in excluded sites was set to zero. The model produced estimates of the probability that a pathogen causes disease at each covariate value (age and enrollment time). We then pooled results to calculate an overall estimate of the etiology fraction in each country for each pathogen, presented as an average (mean) with 95% credible intervals, assuming age and enrollment time distribution represents the diseased population in the country.

## Results

### Enrollment

**Fig 1:**
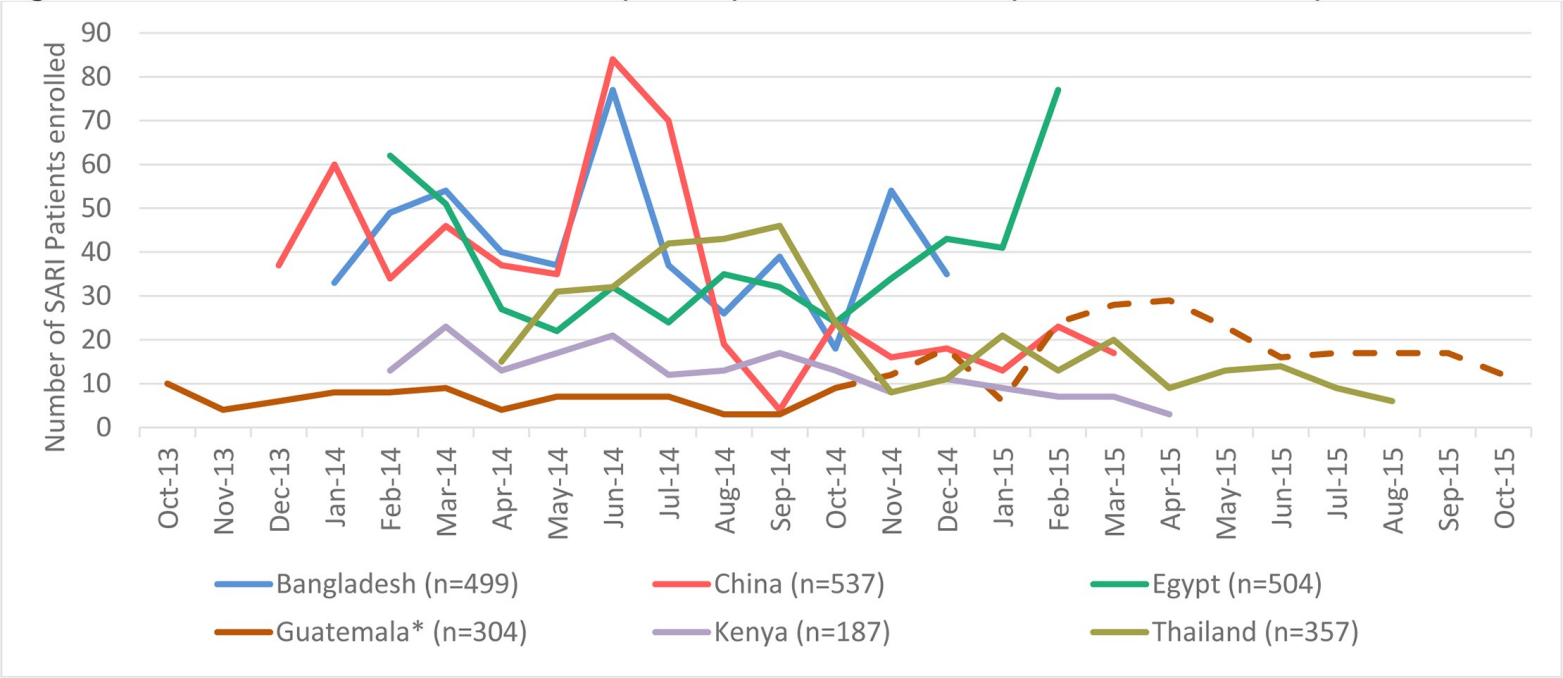
Numbers of severe acute respiratory infection (SARI) patients enrolled by week and site. *Temporary suspension of enrollment for 6 weeks in August-September 2014. **Dotted line indicates time when additional hospital added to surveillance in Guatemala in October 2014.

We enrolled 2,388 patients with SARI and 1,135 asymptomatic adults across all sites from October 2013 through October 2015 (Table 2). Each site enrolled participants for a minimum of 12 months (Bangladesh) and up to 24 months (Guatemala). Sites enrolled all eligible patients for the duration of the study with several exceptions. Bangladesh and Guatemala did not enroll patients who were admitted and discharged during periods when surveillance staff was unavailable (weekends, holidays). China had a temporary suspension of enrollment for six weeks in August and September of 2014. Thailand included 357 SARI patients of 462 who were eligible (77%). Those not included either refused consent or were unable (e.g., incapacitated) to consent. In Egypt and Kenya, we were unable to determine the number of eligible patients not enrolled. Enrollment over time is shown in Fig 1.

**Table 2:** Basic demographic and underlying medical conditions for adults with severe acute respiratory infections (SARI) and asymptomatic adults.

	Bangladesh		China		Egypt		Guatemala		Kenya		Thailand	
	SARI patient (n = 499) %	Asymptomatic adults (n = 198) %	SARI patient (n = 537) %	Asymptomatic adults (n = 216) %	SARI patient (n = 504) %	Asymptomatic adults (n = 209) %	SARI patient (n = 304) %	Asymptomatic adults (n = 174) %	SARI patient (n = 187) %	Asymptomatic adults (n = 121) %	SARI patient (n = 357) %	Asymptomatic adults (n = 217) %
Enrollment period	January 2014-December 2014		December 2013-March 2015		February 2014-February 2015		October 2013-October 2015		February 2014-April 2015		April 2014-August 2015	
Age (years)												
18–49	50.3	50.5	77.1	76.9	56.2	54.1	38.7	37.4	59.4	69.4	22.1	24.4
50–64	26.5	26.3	14.5	14.4	33.7	33.5	27.6	28.2	21.4	16.5	30.3	31.8
65+	23.3	23.2	8.4	8.8	10.1	12.4	33.4	34.5	19.3	14.1	47.6	43.8
Median years	48	49	33	40	45	45	57	54	39	39	64	60
Male	64.3	67.7	57.8	51.9	47.6	56.5	32.2	51.7	32.1	18.2	45.1	36.9
**Medical History**^**a**^												
Current smoker	19.4	22.7	24.9*	45.1	22.0	25.4	6.2*	19.0	7.5	4.1	20.5*	11.1
HIV/AIDS	0	0	0	0	0	0	2.0	0	39.6*	57.9	1.7	0
Asthma	24.5*	0	0.4	0	5.8*	1.0	26.6*	1.2	5.4*	0	15.4*	2.8
COPD/Chronic bronchitis	12.2*	1.0	2.2	1.4	1.6	0	4.6*	0	1.6	0	13.2*	0.5
Diabetes	5.0*	1.0	1.1	1.9	9.5	5.3	17.4*	10.3	2.7	0	17.7	12.0
Malaria	0	0	0	0	0	0	0	0	27.8*	2.5	0	0
Immunosuppression^b^	13.0*	0	0	0	0	0	6.3*	0	2.7*	15.7	5.0*	1.4

^a^ Self-reported medical history with the exception of malaria and HIV/AIDS in Kenya which was documented in medical records

^b^ Examples include: receiving chemotherapy, documented as having an autoimmune disease, use of oral or injection corticosteroids for at least 14 days within 2 weeks of enrollment.

*indicates statistically significant (p<0.05) difference between case and control for medical history variable in that site (based on two-sample chi-square test or Fisher’s exact test).

### Demographics and underlying medical conditions

The median age of SARI patients ranged from 33 (China) to 64 years (Thailand); sex distribution varied by site (Table 2). In most countries, SARI patients more frequently had underlying chronic illnesses, including asthma, chronic obstructive pulmonary disease, diabetes, and immunosuppressive illnesses (examples: receiving chemotherapy, documented as having an autoimmune disease, use of oral or injection corticosteroids for at least 14 days within 2 weeks of enrollment) compared to asymptomatic adults. Self-reported smoking was lower among SARI patients than among asymptomatic adults in China and in Guatemala, while smoking was higher among SARI patients than among asymptomatic adults in Thailand. In Kenya, a high proportion of SARI patients had HIV infection [20] and >25% had a malaria co-infection.

### Clinical characteristics and hospital course of SARI patients

Signs and symptoms varied widely by site (Table 3) except for fever and cough, as required by the SARI case definition. In addition to fever and cough, 9% of SARI patients had only upper respiratory tract symptoms (i.e., sore throat, rhinorrhea); 33% had a discharge diagnosis consistent with an upper respiratory tract infection (URTI) and 47% had a discharge diagnosis consistent with a LRTI. In sites with intensive care unit and mechanical ventilation capacity (Table 1), use of these interventions was low (Table 3). Approximately a third of SARI patients reported antibiotic use prior to hospitalization, but this varied widely by site (Thailand: 7%; Egypt: 74%). Among 1,299 SARI patients with chest x-rays taken (55% of SARI patients), 42% had radiographic findings consistent with pneumonia. SARI patients with positive chest x-rays more often presented with shortness of breath (77% vs. 42%), abnormal breath sounds (79% vs. 57%), and tachypnea (35% vs. 25%) when compared to those with no evidence of pneumonia by chest x-ray (each: *P* <0.05), likely correlating with illness severity. In-hospital case fatality was 2% (range 0% [China] to 7% [Kenya]) and more than 40% of those that died were aged ≥65 years. Among SARI patients discharged alive, the median length of stay was 3 days (range: 1 to 107 days). In Kenya, almost half of the SARI patients had no respiratory illness indicated in hospital discharge records. In China, 25.5% of SARI patients reported only upper respiratory symptoms and 85% were discharged with an URTI diagnosis only.

**Table 3:** Clinical description and hospital course of severe acute respiratory infection (SARI) patients.

	Bangladesh^a^	China	Egypt	Guatemala	Kenya	Thailand	Total%
	(n = 499)	(n = 537)	(n = 504)	(n = 304)		(n = 357)	(n = 2388)
	%	%	%	%	%	%	%
Clinical sign or symptom^b^							
Fever	100	100	100	100	100	100	100
Cough	100	100	100	100	100	100	100
Difficulty breathing	70.1	14.8	19.1	88.2	54.0	65.6	47.3
Upper respiratory tract (URT) symptom^c^	44.3	88.6	70.2	79.3	27.8	44.0	62.9
Sputum production	53.3	61.4	74.4	83.9	57.8	70.6	66.4
Hemoptysis	3.1	1.7	4.6	13.5	4.3	3.9	4.7
Fast respiratory rate	36.1	0.9	24.2	26.6	30.5	29.7	23.5
Abnormal breath sounds	72.1	6.5	59.3	89.5	27.3	64.1	52.4
Measured fever above 38^d^	54.5	88.6	26.8	24.7	19.8	63.6	51.2
Pre-hospitalization antibiotics	22.9	29.8	73.8	17.8	19.2^e^	7.4	32.2
Length of stay in days (median, range)^f^	3 (1–30)	3 (1–33)	4 (1–25)	4.5 (1–107)	4 (1–44)	3 (1–32)	3 (1–107)
Days from illness onset to specimen collection (mean, range)	3.8 (1–7)	1.9 (0–7)	4.6 (0–7)	6 (0–10)	4.5 (1–11)	2.6 (0–7)	3.6 (0–11)
Intensive care unit (ICU)^g^	0	0.9	2.2	3.6	3.6	1.0	1.8
Ventilator^6^	0	0.4	0.6	7.2	NA	3.9	2.1
Chest X-ray obtained	56.1	12.2	56.8	88.1	74.9	73.1	54.5
X-ray findings consistent with pneumonia	33.2	47.7	17.1	64.8	37.9	57.9	42.3
X-ray findings not consistent with pneumonia	66.8	49.2	82.9	18.0	44.3	25.7	48.7
Unknown	0.0	3.1	0.0	17.2	17.9	16.5	8.9
Outcome^h^							
Discharged	94.4	99.6	89.7	83.6	87.7	87.1	92.2
Died^i^	2.4	0	1.2	6.3	7.0	1.4	2.3
Discharge Diagnosis							
Acute respiratory infections							
Both URTI^j^ and LRTI^k^	NA	0.2	0.6	0.0	0.5	0.3	0.3
LRTI only	NA	13.2	55.4	70.1	49.7	64.4	46.9
URTI only	NA	85.3	24.8	1.3	1.6	9.2	33.0
Chronic respiratory illness only^l^	NA	0.9	7.3	17.8	1.1	7.0	7.2
No respiratory illness	NA	0.4	11.9	10.9	47.1	19.0	13.3

^a^ Bangladesh did not collect discharge diagnosis information.

^b^ Fever and cough were included in the SARI Patient definition.

^c^ Sore throat and/or runny nose.

^d^ Maximum temperature recorded prior to hospitalization, at admission, at enrollment, or recorded in the first 24 hours of hospital admission.

^e^ Missing more than 10% of responses from SARI patients.

^f^ SARI Patients that died or were transferred to another facility are excluded.

^g^ Not all hospitals have ICU or ventilators; percentages calculated based on the number of SARI Patients at hospitals with ICUs and/or ventilator capacity.

^h^ SARI Patients that did not die or were not discharged were classified as transferred to another facility or left against medical advice.

^i^ Case fatality rate: 18–49 years of age, 1.1%; 50–64 years of age, 2.9%; 65+ years of age, 4.4%.

^j^ Upper respiratory tract infection and otitis.

^k^ Lower respiratory tract infection, pulmonary tuberculosis, PCP pneumonia, bronchitis, pneumonia, empyema.

^l^ COPD and asthma.

### Estimated SARI incidence rates

In total, 2,219 cases (92.9%) were included in incidence rate calculations (Table 4); some cases were from areas for which the catchment population was unknown. Annual incidence of SARI ranged from 15 in Bangladesh to 384 in Kenya (per 100,000 population). Rates increased with age and were highest among adults aged ≥65 years in all sites.

**Table 4:** Severe acute respiratory infection incidence rates, by participating site and age group.

	18–49 years old			50–64 years old			65+ years old			All Adults		
	Case	Person-years	Rate per 100,000 (LCL-UCL)	Case	Person-years	Rate per 100,000 (LCL-UCL)	Case	Person-years	Rate per 100,000 (LCL-UCL)	Case	Person-years	Rate per 100,000 (LCL-UCL)
**Bangladesh**^**a**^	226	2,441,487	9 (5–18)	132	420,740	31 (22–45)	116	218,507	53 (41–69)	474	3,080,734	15 (9–25)
**China**	414	1,129,980	37 (27–51)	78	202,596	39 (28–53)	45	85,225	53 (40–69)	537	1,417,801	38 (28–52)
**Egypt**^**1**^	271	102,251	265 (235–299)	170	24,069	706 (656–760)	51	6,542	780 (727–836)	492	132,862	370 (334–410)
**Guatemala**^**b**^	65	567,486	11 (6–20)	41	105,132	39 (28–53)	62	71,172	87 (71–107)	168	743,790	23 (15–34)
**Kenya**^**c**^	48	16,143	297 (265–333)	20	3,327	601 (555–651)	18	2,906	620 (573–670)	86	22,376	384 (348–425)
**Thailand**^**d**^	112	236,434	47(36–63)	135	73,343	184 (159–213)	215	37,879	568 (523–616)	462	347,656	133 (112–158)

^a^ In Bangladesh and Egypt, population data was available only for 20–49 years of age and rates were calculated using SARI Patient patients 20 and older.

^b^ Restricted to Santa Rose and Quetzaltenango study sites.

^c^ SARI Patients and population data restricted to Karemo region for Siaya District Hospital and population based infectious disease surveillance area for Lwak Mission Hospital.

^d^ Thailand incidence calculations included SARI patients that were eligible for study enrollment but did not consent for study participation.

### Pathogen detections prior to modeling

**Fig 2:**
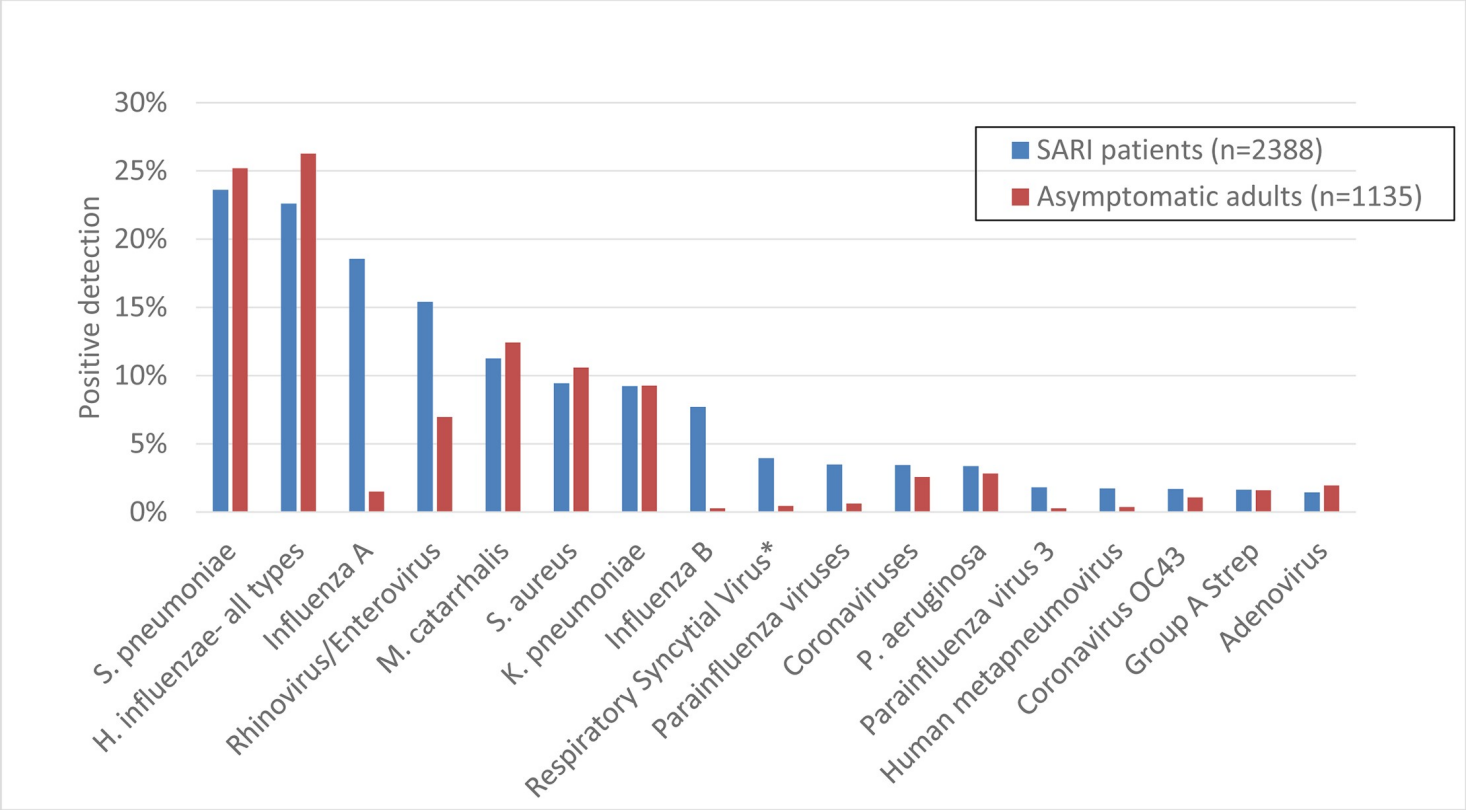
Percentage of severe acute respiratory infection (SARI) patients and asymptomatic adults with positive detections by pathogen, all sites. *RSV excluded in China due to contamination. Pathogens comprising <1% of detections are not shown.

One or more pathogens were detected in specimens from 77% of SARI cases and 67% of asymptomatic adults (Fig 2). The frequency of positive detections among SARI patients and asymptomatic adults and resulting odds ratios by site are reported in S1 Table. The most commonly detected pathogens among both SARI patients and asymptomatic adults were *S*. *pneumoniae* (24% and 25%, respectively) and *H*. *influenzae* (23% and 26%). Pathogens more commonly detected among SARI patients than asymptomatic adults included influenza A (18.6% of SARI patients vs. 1.5% of asymptomatic adults), influenza B (7.7.% vs. 0.3%), rhino/enteroviruses (15.4% vs. 7.0%), and respiratory syncytial virus (RSV) (4.0% vs. 0.4%). Thailand, Guatemala, and Bangladesh had the highest prevalence of RSV detections among SARI patients (9.0%, 4.3%, and 3.4%, respectively). Two or more pathogens were detected in 39.5% of SARI patients and 30.1% of asymptomatic adults (S1 Fig). A bacterial pathogen was detected in approximately half of asymptomatic adults. SARI patients more commonly had only viral detections (22.3%) or both viral and bacterial detections (27.2%) when compared to asymptomatic adults (5.6% and 7.6% detected, respectively) (S2 Fig). There was no difference in the frequency of ICU admission and the outcome at discharge between patients with a single pathogen detected and patients with multiple pathogens detected. Additional figures describing influenza and RSV circulation are available in S3–S5 Figs.

Estimated true positive rates (the proportion of SARI cases with an infection that is caused by a pathogen that test positive for that pathogen) ranged from 63% for human metapneumovirus to 91% for *H*. *influenzae (all types)* (S3 Table).

### Modeling results: Attributions of pathogens to etiology

**Fig 3:**
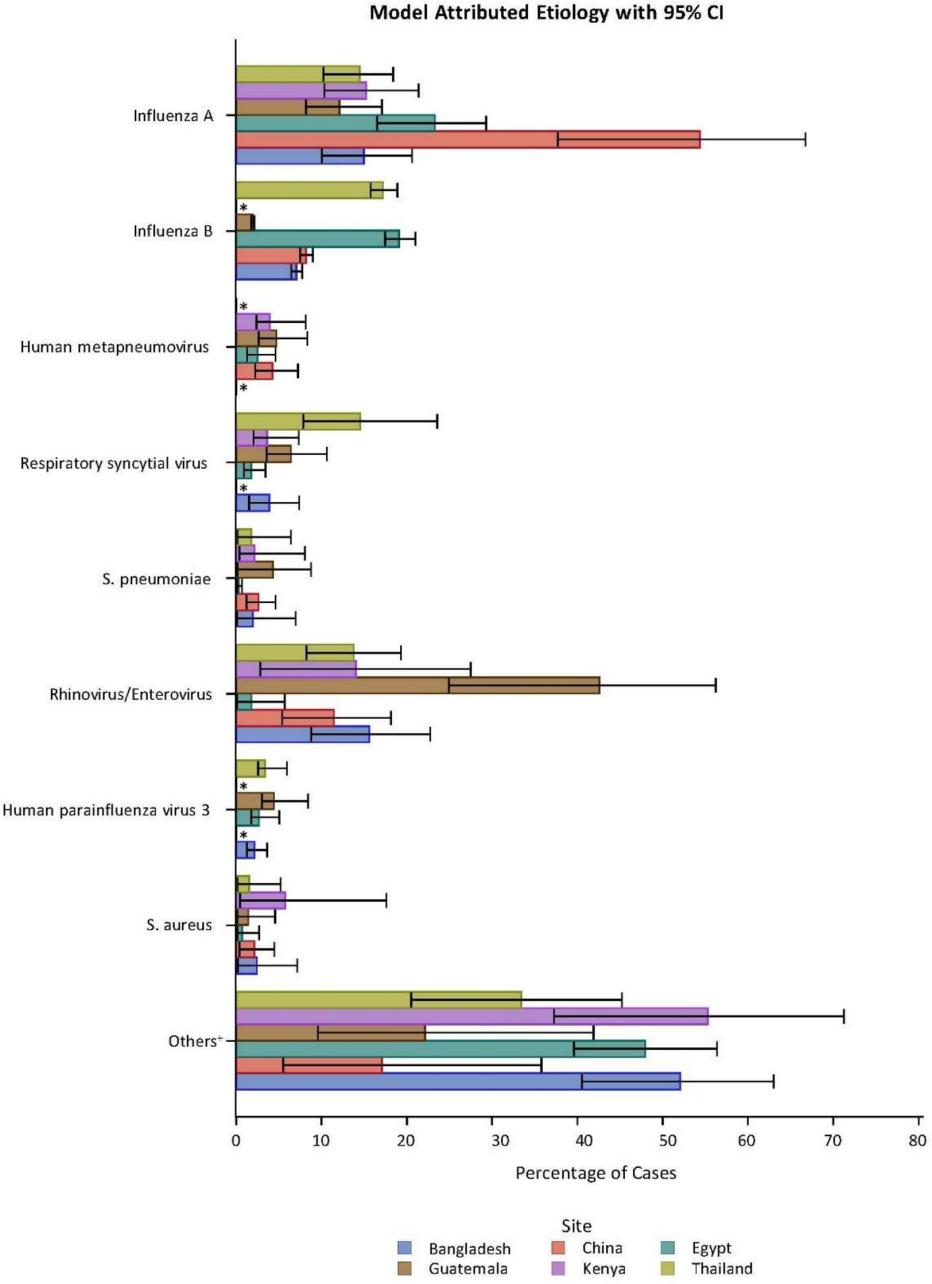
Estimated attributed etiology by pathogen and site*. Bar represents 95% credible interval. *Indicates countries with no etiology attributed to pathogen. +Percentage that could not be attributed to one of these eight pathogens, including pathogens not included on the TAC.

Results of Bayesian latent class modeling of the attribution of etiology are summarized by site in Fig 3 and S2 Table. The most commonly attributed pathogens across all sites were influenza A, influenza B, and rhinovirus/enterovirus (Fig 3). Attribution of pathogens varied by site, however. Influenza A was attributed as the etiology of >12% of cases in all sites and was estimated to be the etiology of 54% of SARI cases in China and 23% of all SARI cases in Egypt. Proportion of SARI cases attributed to influenza B ranged from 2% in Guatemala to 17% and 19% in Thailand and Egypt, respectively; influenza B was not detected in Kenya. Rhinovirus/enterovirus was the estimated etiology in at least 11% of SARI cases in five sites, including 11.5% of SARI cases in China and 43% in Guatemala (S2 Table). Human metapneumovirus comprised 2.5%-4.7% of SARI cases in four sites. RSV was a commonly attributed etiology in Thailand (14.6%). Several bacterial species were identified as SARI etiologies: *Klebsiella pneumoniae* (5.2% in Bangladesh, 5.9% in Kenya); *Moraxella catarrhalis* (6.1%, Thailand); *Mycoplasma pneumoniae* (2.3%, China); *Pseudomonas aeruginosa* (3.7% in Guatemala, 4.8% Bangladesh); and *S*. *pneumoniae* (0.3% in Egypt, 4.3% in Guatemala). An etiology was attributed to a pathogen on TAC in the majority of cases, ranging from 57% in Egypt to more than 93% in China. We were unable to attribute etiology for the subset of patients with chest x-ray confirmed pneumonia or for those with LRTI diagnosis due to small numbers.

## Discussion

This is the first multi-country study using comparable methods in geographically diverse settings to describe the distribution of 29 pathogens among adults with SARI and to estimate their contributions to SARI etiology. TAC technology enabled detection of multiple viral and bacterial pathogens from NP/OP specimens collected from participants in six countries. We found a high prevalence of detection of bacteria in the upper respiratory tract of both SARI patients and asymptomatic adults. Through modeling, the most common etiologies of SARI among participating adults were viruses. Bacterial infections were estimated to cause 5% to 20% of SARI illnesses. Knowledge of pathogen distribution in each site may aid future investigations of respiratory outbreaks of unclear etiology by providing background rates for commonly circulating pathogens among adults.

We documented the burden of SARI among adults in countries that differ by multiple factors that likely impact pathogen distribution, such as seasonality of respiratory pathogens, climate, and access to vaccines. Other studies have reported incidence rates of SARI in persons aged ≥5 years ranging from 67 per 100,000 in Guatemala to 310 per 100,000 in Kenya [34, 35], similar to the rates we found in our analysis. The relatively low SARI incidence rates noted in Bangladesh have been previously reported and may be due to differences in health care seeking behavior or in health care providers’ thresholds for hospitalization of an ill person [36]. Age distribution, sex, and severity of disease of SARI patients enrolled varied by country, which may be due to differences in care seeking behavior, healthcare decision-making, access to care, population age-structure, or socioeconomic status, resulting in differences in pathogen detections and etiology attribution.

The most commonly detected pathogens among enrolled adult patients were *S*. *pneumoniae*, *H*. *influenzae*, influenza A, rhinovirus/enterovirus and *M*. *catarrhalis*—each found in at least 10% of SARI patients. After modeling was conducted, *S*. *pneumoniae* and *H*. *influenzae* were not considered to be the most common causes of SARIs due to the high number of detections also seen in asymptomatic adults, likely indicating colonization. The pathogens most frequently identified as the etiology of SARI included influenza A and B, rhinovirus/enterovirus, and RSV; influenza accounted for 14–63% of SARI cases. Our findings highlight the likelihood that global use of influenza vaccines—and potentially RSV vaccine in the future—could significantly reduce disease burden among adults [37].

Several limitations preclude accurate determination of the etiology of SARI. The principal challenge is reliance on specimens taken from the upper respiratory tract to model etiology of infections in the lower respiratory tract [38]. More informative specimen types, such as lower respiratory specimens, are more invasive and often challenging to collect, while less invasive specimens—such as urine and upper respiratory tract specimens—have diagnostic limitations. For example, urine antigen detection is currently limited to two pathogens (pneumococcus, Legionella). Also, blood culture is highly specific but insensitive for most pathogens. While use of PCR is highly sensitive for detecting multiple respiratory pathogens, including only upper respiratory tract specimens will result in missed detection of some pathogens which are rarely found here (e.g., Legionella) and will detect other pathogens that are present in the upper respiratory tract as colonizers but not as the infectious agent of the lower respiratory tract. Secondly, although convenient, the use of the WHO SARI case definition does not capture all severe respiratory illnesses. The SARI definition was developed for influenza surveillance and therefore maximizes detection of influenza infections; it does not capture pneumonia cases that present without fever and includes some individuals without lower respiratory tract infections who are hospitalized with severe upper respiratory infections, as found in Kenya and China, based on discharge diagnosis. Enrollment of these individuals may have contributed to challenges with attributing etiology in this study and may be related to improper use of the case definition, lack of appropriate documentation of the patient discharge diagnosis in the chart, or presentation of individuals with acute respiratory infections who are actually hospitalized for other causes. Challenges with acute respiratory disease case definitions have been well documented [39–41] and likely contribute to difficulties in attributing specific pathogens as the cause of disease. Additionally, different sources of asymptomatic adults could have diverse impacts on the estimated etiology proportion estimations. In our study we used a variety of sources of controls, each with different, unquantified, potential for bias. In general, the most common direction of bias would likely result in an underestimation of the etiology proportion for a particular pathogen.

We excluded asymptomatic adults with respiratory symptoms in the seven days prior to enrollment; however, viral shedding of some viruses has been documented for much longer than seven days, particularly in individuals with underlying medical conditions [42–44]. Therefore, we may have detected some pathogens from a prior respiratory illness in asymptomatic adults. Also, there is the possibility of detecting a pathogen in the latent stage just before an individual becomes symptomatic. Comparing detection of pathogens known to colonize the upper respiratory tract in patients and asymptomatic adults tends to disregard the contributions of these pathogens to disease; for example, *Streptococcus pneumoniae* has been shown to cause a substantial proportion of pneumonia when appropriate specimens and additional diagnostics such as urine antigen test are used [7, 45, 46]. Additionally, URTIs caused by viral pathogens can predispose people to secondary bacterial infections, including infections caused by bacteria often associated with asymptomatic colonization. Comprehensive etiology studies incorporate lower respiratory specimens, blood and urine [41], although these more informative specimen types are rarely collected, particularly in resource limited settings.

There are multiple possible explanations for the proportion of SARI cases in which a pathogen was not detected on TAC. First, for detection of several important bacterial causes of pneumonia, such as *M*. *tuberculosis* and *Legionella*, NP and OP swabs are not ideal specimens. Second, antibiotic use prior to hospitalization may have reduced bacteria in the naso- and oropharynx of some participants, particularly in Egypt where antibiotic prior to hospitalization of SARI patients was over 70%. Third, some potential SARI pathogens (e.g., *Coxiella burnetii*, melioidosis, fungal infections) are not included on TAC. Finally, timing of specimen collection may result in missed detection of pathogens for which clinical disease presentation lags behind actual infection.

Our findings can assist both public health and clinical providers interpret the results of multi-pathogen diagnostic tests, which are increasing available for use, by providing background rates of detection. Also, the prevalence of both viral and bacterial pathogens—and often two or more pathogens—in the upper airways of asymptomatic adults should remind both public health practitioners and clinical providers that the simple detection of respiratory pathogens (excluding influenza and RSV) in the upper airways should be interpreted with care, as they may not be the etiology of infection. Other supportive evidence should be taken into account when interpreting the significance of detection of each pathogen and when developing patient treatment plans. This includes all available clinical data (signs, symptoms and available laboratory findings) and the epidemiologic, demographic and risk factor data found among similarly affected persons in respiratory outbreak investigations.

A key strength of our study was the use of a quantitative model based on the partially latent class model developed for the PERCH study—a multi-site international case-control study of etiology of pneumonia among children under five years of age utilizing multiple specimen types [47]. It models the pathogen etiology directly and has demonstrated success by improving accuracy when compared to a more traditional approach based on PAR [33, 48]. This novel model was then further modified to allow the etiology attribution, as well as the false positive rates, to vary by covariates (e.g., age group, season) through a non-parametric Bayesian approach, as applied to the Aetiology of Neonatal Infections in South Asia and Sepsis Aetiology in Neonates in South Africa studies [13, 14, 49]. Our study is the first application of this modeling approach in an adult population and contributes to the understanding of SARI etiology. This modeling approach allows for site-specific differences and assumes etiology proportions and carriage rates vary by site.

Despite its theoretical advantages and successful application in other studies [47–49], the modeling approach has several limitations. The model assumes that carriage of pathogens is independent, given the actual infection status and specific covariates. Additionally, the implication of co-detection of multiple pathogens in NP/OP specimens from SARI patients is unclear, and the implemented model cannot estimate the proportion of SARI cases in which more than one pathogen contributed to disease. This highlights the need for further research to understand the significance of co-detections. An additional assumption of the model is that infection with one pathogen does not change the likelihood of carriage of another pathogen. Some organisms, such as *S*. *pneumoniae*, which are both frequent colonizers and common etiologies of pneumonia, have demonstrated that convergence and accuracy of the model are improved by obtaining data from more than one type of diagnostic test (e.g., culture and non-culture methods) on multiple specimen types [13, 33, 47–49].

## Conclusions

Use of multi-pathogen diagnostics among adults with SARI provided a global snapshot of the distributions of pathogens found in upper respiratory tract specimens from SARI patients and compared the concurrent distributions of these pathogens across six countries. Frequent detection of multiple pathogens in upper respiratory tract specimens, lack of lower respiratory tract specimens, and frequent pathogen co-detection made etiology attribution difficult. However, use of advanced latent class methodology highlighted the importance of several viruses as the etiology of SARI, including influenza, RSV, and rhino/enterovirus, and identified *S*. *pneumoniae* as the most commonly attributed bacterial cause. The high proportion of SARI cases attributed to influenza and RSV indicates that public health interventions, such as regular use of seasonal influenza vaccine and development and introduction of RSV vaccine, would likely reduce burden of SARI in this population. The findings of this study also provide background rates of the detection of multiple viral and bacterial pathogens among ill adults in these countries, enabling.

## Supporting information

S1 File Laboratory methods. (DOCX)

S2 File Brief overview of the statistical methodology. (DOCX)

S1 Fig Number of pathogens detected per person among severe acute respiratory infection (SARI) and asymptomatic adults. (DOCX)

S2 Fig Percent of bacterial and viral detections among severe acute respiratory infection (SARI) patients and asymptomatic adults. (DOCX)

S3 Fig Number of severe acute respiratory infection (SARI) patients in which influenza A was detected by site and month. (DOCX)

S4 Fig Number of severe acute respiratory infection (SARI) patients in which influenza B was detected by site and month. (DOCX)

S5 Fig Number of severe acute respiratory infection (SARI) patients in which RSV was detected by site and month. (DOCX)

S1 Table Comparison of frequency of pathogens detected among severe acute respiratory infection (SARI) patients and asymptomatic adults by site. (DOCX)

S2 Table Estimated attributed etiology of severe acute respiratory infection (SARI) patients by pathogen and site. (DOCX)

S3 Table True positive rate* of the TAC assays. (DOCX)

S4 Table Percentage of severe acute respiratory infection (SARI) patients and asymptomatic adults in which pathogens were detected. (DOCX)

S1 Dataset (XLSX)
