# Supplementary material for: The epidemiology and estimated etiology of pathogens detected from the upper respiratory tract of adults with severe acute respiratory infections in multiple countries, 2014–2015
Source: PLoS One. 2020 Oct 19;15(10):e0240309. doi: 10.1371/journal.pone.0240309 (PMC7571682; doi:10.1371/journal.pone.0240309)
Supplement: S1 File — (DOCX) [file pone.0240309.s001.docx]

**S1 File.** Laboratory methods

Combined nasopharyngeal and oropharyngeal specimens were transported and stored at 4°C. Specimens were either tested within 72 hours of collection or stored at -70°C until testing could be completed. TAC testing was performed on the ViiA 7 Real-time PCR System (ThermoFisher Scientific) using the AgPath-ID one-step kit (Thermo Fisher Scientific).^15^ Using site-specific extraction instruments or kits, participating sites used between 100 µL and 200 µL of input specimen volume and eluted in 100 µL. A master mix of 50 µl of AgPath-ID 2x reaction buffer and 4 µl of enzyme was prepared for each specimen, and 46 µl of extracted total nucleic acid was added to the mix for a final volume of 100 µl. The 100 µl mix was dispensed into the loading well of the card and centrifuged twice on a Sorvall Legend T (Thermo Fisher Scientific) at 1,200 rpm to ensure equal loading of the wells. Six specimens, one positive control^32^ and one negative control were included on each card. Laboratorians from all sites were trained to ensure standardized use of the TAC platform and proficiency testing panels were completed by all laboratorians. TAC run files were sent to CDC for review to verify accuracy of the analysis.

*Interpretation of TAC results*

The combined nasopharyngeal and oropharyngeal specimen was defined as positive for the presence of the target nucleic acid for a pathogen if all specimen and assay controls on each card performed as expected, and at least one assay well was positive (i.e., indicated by a sigmoidal shaped curve at any Ct value) for the given target. The specimen was defined as negative for the presence of the target nucleic acid if all controls for the TAC and specimen worked as expected, and no wells were positive for the given target. Indeterminate results were assigned in the event of a molecular control failure (indicated by negative human RNaseP serving as a specimen quality control, negative target positive control, or any detections in the non-template control), a Ct value >35 for *Legionella pneumophila* or *Bordetella pertussis*, or a non-sigmoidal florescence amplification curve. Where possible, specimens were retested if any of the controls failed. Due to the cross reactivity of the rhinovirus and enterovirus assays, the results were assigned as rhinovirus/enterovirus positive if either target was positive, and a rhinovirus/enterovirus negative if both targets were recorded as negative.
