# Supplementary material for: The epidemiology and estimated etiology of pathogens detected from the upper respiratory tract of adults with severe acute respiratory infections in multiple countries, 2014–2015
Source: PLoS One. 2020 Oct 19;15(10):e0240309. doi: 10.1371/journal.pone.0240309 (PMC7571682; doi:10.1371/journal.pone.0240309)
Supplement: S2 File — (DOCX) [file pone.0240309.s002.docx]

**S2 File.** Brief overview of the statistical methodology

1. **Definitions of etiology proportion based on laboratory test:**

For one specific pathogen *k* and a corresponding binary laboratory diagnostic test $T_{k}$, the etiology proportion, or etiology fraction of the pathogen to a disease can be defined from two different perspectives.

The first perspective considers exposure to the pathogen as a risk factor to develop the disease and the exposure is indicated by the laboratory test results. In other words, a positive test result would indicate the subject had been exposed to the pathogen. Then the etiology proportion of the pathogen to the disease is defined as the proportion of diseased cases being prevented should the pathogen exposure be removed completely from the study population, i.e., the population attributable risk of the pathogen to the disease:

$PAR= \frac{l_{e}-l_{u}}{l_{e}}$ [1]

An equivalent expressions of PAR is:

$PAR= \frac{P_{e}\left( {RR}_{e}-1 \right)}{1+P_{e}\left( {RR}_{e}-1 \right)}$ [2]

where $P_{e}$ is the prevalence of the exposure and ${RR}_{e}$ is the relative risk of disease due to the exposure.

The second perspective extends the laboratory test, which actually only indicates if the pathogen appears in the collected specimen, into a pseudo test for disease etiology with following accuracy measures:

$$\theta=P\left( laboratory result positive \right|cases caused by the pathogen)$$

$$\delta=P\left( laboratory result positive \right|cases not caused by the pathogen)$$

Then it considers the population of cases as a mixture two sub-populations: those caused by the pathogen and those from other causes. The proportion of the first subpopulation is the etiology proportion and can be expressed as:

$EF=\frac{p_{p}-\delta}{\theta-\delta}$ [3]

here $p_{p}$ is the proportion of having positive laboratory test results among all cases. The two quantities $\theta$and $\delta$ are called true positive rate (TPR) and false positive rate (FPR) respectively.

Although the two perspectives seem very different, it can be shown that under a simple condition:

$\delta=\theta\times P_{e}$ *[4]*

the two perspectives defines the same etiology proportion.

To understand what might be implied in equation [4], let’s consider a simple, yet common infectious disease development process in which exposure to pathogen will first result in a carriage status before developing into disease. Suppose the pathogen will appear in the collected specimen for subjects in either carriage or disease status, then the true positive rate $\theta$ is the laboratory test’s sensitivity. Hence the left side of equation [4] is the probability of being tested positive for subjects in the study population. Accordingly, equation [4] will be approximately true if only a small proportion of subjects in carriage status will develop into disease. In other words, if removing diseased cases will not modify carriage proportion $C_{e}$ substantially, then the two definitions of pathogen etiology proportions are approximately the same.

To actually calculate etiology proportions defined by either of the perspectives, we need a sample from non-diseased population to estimate both $P_{e}$ and ${RR}_{e}$ in the PAR formula (equation [2]), and the false positive rate $\delta$ in the mixture model formula (Equation [3]). They also require value of the true positive rate $\theta$, although in regular use of PAR it always implies that $\theta$ = 100%. For that reason, the actual calculation of PAR is always biased.

Both of the two perspectives can be extended to multiple pathogens situations, namely to define etiology proportions of multiple pathogens simultaneously. The extension of the PAR approach is to consider each pathogen’s laboratory test results as an indication of being exposed to the pathogen, which in turn being considered as a risk factor for developing disease. Then a multiple logistic regression can be applied to obtain “adjusted” odds ratios for the pathogens, and then replaced the relative risk in equation [2] by the odds ratio to calculate the “adjusted” PARs. Unfortunately, such an ad hoc approach loses the simple epidemiology interpretations of etiology proportions and often causes inconsistent results such as the sum of individual PAR might be larger than 100%, and sometime the “adjusted” odds ratio might be less than 1.0 resulting in negative PARs. More ad hoc adjustments such as removing pathogens with odds ratio less than 1.0 will further cause logical interpretations of PARs. Further, as we mentioned earlier, the PAR approach assumes 100% sensitivity of the laboratory tests which is often untrue.

On the other hand, the extension of the mixture model approach to multiple pathogens are much more straightforward. The partially-Latent Class Model (pLCM) developed by Wu. et al.^1,2^ for the Pneumonia Etiology Research for Child Health (PERCH)^3^ is a recent development in the area. With an extra assumption on the relationships between different laboratory tests (conditional independence assumption), pLCM can be used to estimate the etiology proportions even if the true positive rates of the laboratory tests are unknown, through a Bayesian approach. We will introduce Wu’s basic pLCM approach in section 2 below. Then in section 3, we further extend the basic model to include covariates, namely from a simple latent class model into a more complicated latent class regression model. The detailed implementation of the method will be summarized in section 4.

1. **The basic pLCM (partially-Latent Class Model):**

Suppose there are $K$ targeted pathogens (30 on the TACs) each with one diagnostic test $T_{k}, k=1,\ldots K$ that produces binary (positive vs negative, or 1 vs. 0) test result $y_{ik}$ for case$i, i=1,\ldots,N$. We add one extra class (referred to throughout as ‘Other/None’) for other pathogenic or non-pathogenic causes and code it as class$(K+1)$. If we assume each individual case has only one etiology cause from the $K+1$ classes, then the population of cases can be considered as a mixture of subpopulations with etiology$k=1,\ldots,K, K+1$. Let $Z_{i}$ be the true (unobserved) etiology of case$i, i=1,\ldots,N$, then the objective of the model is to estimate probability$\pi_{k}=P\left( Z_{i}=k \right), k=1,2,\ldots,K,K+1$, using the observed binary test results$\left\{ y_{ik}, k=1,\ldots,K;i=1,\ldots,N \right\}$. Here $0\leq\pi_{k}\leq1$ and$\sum_{k=1}^{K+1} \pi_{k}=1$. We refer to $\pi_{k}$ as pathogen proportion throughout.

pLCM expresses the probability of observing $\left\{ y_{ik} \right\}$ through a linear mixture model with ${\{\pi}_{k}\}$ as the mixing coefficients. By applying the regular conditional independence assumption for such linear mixture class models with unknown (latent) classes, and a further assumption that the probability of test $T_{k}$ to produce positive test result depends only on whether pathogen $k$ is the true etiology of the tested case, the linear mixture can be simplified as:

$f\left( y_{ik}, k=1,\ldots,K;i=1,\ldots,N \right)= \prod_{i=1}^{n} (\sum_{k=1}^{K} \pi_{k}\theta_{k}^{y_{ik}}\left( 1-\theta_{k} \right)^{1-y_{ik}}\prod_{j\neq k} \delta_{j}^{y_{ij}}\left( 1-\delta_{j} \right)^{1-y_{ij}}+ \pi_{K+1}\prod_{j} \delta_{j}^{y_{ij}}\left( 1-\delta_{j} \right)^{1-y_{ij}})$ [5]

Here parameters $\theta_{k}=f\left( y_{ik}=1 \right| true etiology=k)$ and $\delta_{k}=f\left( y_{ik}=1 \right| true etiology \neq k)$ are called the True Positive Rate (TPR) and False Positive Rate (FPR) respectively for test$T_{k}, k=1,\ldots K$.

The parameters in Equation [5] can be estimated under a Bayesian analysis framework using conjugate priors for the parameters, for example, (K+1)-class Dirichlet distributions for the pathogen proportions and Beta distributions for the TPRs and FPRs.

The performance of pLCM was evaluated through simulation studies. Knoll et al demonstrated that pLCM outperforms the regular etiology fraction method based on population attributable risk (PAR) .^4^ Further, they showed that with a large sample of healthy subjects , an adequate number of cases with confirmed pathogen infection (such as through blood culture isolation), and prior knowledge of the TPRs associated with diagnostic tests used for a subset of pathogens, the basic pLCM will usually produce reliable pathogen proportion estimates. More extensive simulation experiments by Shang et al showed that even with non-informative priors, pLCM can still estimate pathogen proportions reliably, especially for the class of “Others/None”, if at least a few pathogens are tested by more than one laboratory test.^5^

1. **An extension of pLCM with covariate dependent pathogen proportions and false positive rates**

The TAC tests employed in our study were developed by extensive testing of the target pathogens and nearest neighbors to ensure high laboratory specificity.^6^ Hence a positive test result almost certainly indicated that the pathogen was present in the collected specimen. Since we assume a single etiology cause for each case, positives for non-etiological pathogens indicate pathogen carriage. Because pathogen carriage rates likely change over many covariates such as location (study site), season (enrollment date) and age, false positive rates in pLCM should vary similarly. However, if false positive rates vary by covariates, an identical set of test results may have different etiologic meaning at different covariate values. Consequently, pathogen proportions cannot be determined by test results alone but should also vary by covariates. Thus we extended the basic pLCM to the following model:

$f\left( \tilde{Y}_{1}, \cdots, \tilde{Y}_{n} \right)= \prod_{i=1}^{n} (\sum_{k=1}^{K} \pi_{k}^{x_{i}}\theta_{k}^{y_{ik}}\left( 1-\theta_{k} \right)^{1-y_{ik}}\prod_{l\neq k} {(\delta_{l}^{x_{i}})}^{y_{il}}\left( 1- \delta_{l}^{x_{i}} \right)^{1-y_{il}}+ \pi_{K+1}^{x_{i}}\prod_{l} {(\delta_{l}^{x_{i}})}^{y_{il}}\left( 1- \delta_{l}^{x_{i}} \right)^{1-y_{il}})$ [2]

Here $(x_{1},\ldots,x_{n})$ are the observed values of the covariates for the *n* cases. $\tilde{\Pi}^{x}=\left( \pi_{1}^{x}, \cdots,\pi_{K}^{x},\pi_{K+1}^{x} \right)$ and $\tilde{\Delta}^{x}=\left( \delta_{1}^{x}, \cdots,\delta_{K}^{x} \right)$ are the pathogen proportion distributions and false positive rates for any $x \in\mathcal{X}$. Notice that we hold the true positive rates $\Theta=\left( \theta_{1},\ldots,\theta_{K} \right)$ constant across covariate levels because we assume that infection by a pathogen implies presence of the pathogen in the respiratory tract of cases.

Allowing false positive rates to vary by covariates is not only necessary to address known variation in pathogen carriage, but also alleviates violations of the conditional independence assumption in pLCM. This is because covariates are often confounders for co-carriage of some pathogens. Through adjusting the confounding effect, dependence between test results for co-carried pathogens may weaken, both locally and globally.

A Bayesian Kernel Model approach was developed to estimate parameters in the extended model as expressed by Equation [2]. For each data point $x$ in the domain of the covariates, we assume pathogen proportion $\tilde{\Pi}^{x}=\left( \pi_{1}^{x}, \cdots,\pi_{K}^{x},\pi_{K+1}^{x} \right)$ to have a Dirchlet prior with parameters$\left( e_{1}^{x0}, \cdots,e_{K}^{x0},e_{K+1}^{x0} \right)$, and the false positive rates $\delta_{k}^{x}, k=1,\ldots,K$ to have Beta priors with parameters$\left( c_{k}^{x0},d_{k}^{x0} \right),k=1,\ldots,K$. We also assume the posterior distributions of the parameters can be approximated by the same type of distributions and thus use them as the sampling distributions in the next iteration of the Gibbs Sampler. The parameters of the sampling distributions are updated by the following equations:

$e_{k}^{x}=e_{k}^{x0}+\sum_{i=1}^{N} z_{ik}\times d(x,x_{i}), k=1,\ldots,K, K+1$ [3]

$c_{k}^{x}=c_{k}^{x0}+\sum_{i=1}^{N} y_{ik}\times z_{i\left( K+1 \right)} \times d(x,x_{i}), k=1,\ldots,K$ [4a]

$d_{k}^{x}=d_{k}^{x0}+\sum_{i=1}^{N} {(1-y}_{ik})\times z_{i\left( K+1 \right)} \times d(x,x_{i}), k=1,\ldots,K$ [4b]

Here $Z_{i}= \left( z_{i1},z_{i2},\ldots,z_{iK},z_{i(K+1)} \right)$are the imputed pathogen (latent) classes for the etiology of case *i* from the previous iteration of the Gibbs sampler. $z_{ik}$ takes value 0 or 1 only, and $\sum_{k=1}^{K+1} z_{ik}=1$. The quantity $d\left( x, x_{i} \right)$ measures the contribution of case *i* at $x_{i}$ to data point$x$, such that$0\leq d\left( x, x_{i} \right)\leq1$, $\max_{x} d\left( x, x_{i} \right)=d\left( x_{i},x_{i} \right)=1$, and $d\left( x, x_{i} \right)$ decreases as the distance between $x$ and $x_{i}$ increases. For discrete covariates, $d\left( x, x_{i} \right)$ takes value 1 or 0 depending whether $x_{i}$ and $x$ share the same covariate values. For continuous covariates

$d\left( x,x_{i} \right)=C \times K\left( x \right|x_{i}, h)$ [5]

Here $K\left( x \right|x_{i}, h)$ is a Gaussian density function with mean at $x_{i}$and standard deviation$h$. $C$is a constant to make sure that $d\left( x_{i},x_{i} \right)=1$.

The prior distributions of parameters are constructed by adding (K+1) pseudo cases to the study population (one pseudo case per pathogen class). Each pseudo case has equal probability to be positive or negative for each of the laboratory tests. Hence, the prior distribution for the true positive rates, which are invariant by covariates, are the Jeffrey non-informative prior for binary events, or Beta (0.5, 0.5). When covariates are considered, each pseudo case will be considered as a probability density function uniformly distributed on the domain $\mathcal{X}$ of the covariates. Its overall contribution to a data point $x\in\mathcal{X}$ is then:

$d\left( x \right)= \frac{1}{\left| \mathcal{X} \right|}\int_{t \in\mathcal{X}} d\left( x, t \right)dt$ [6]

Here we assume the domain of the covariates is finite and $\left| \mathcal{X} \right|$ is its volume (area). Hence, the prior for the pathogen distribution at data point $x$ will be $Dirichlet(d\left( x \right), d\left( x \right), \ldots, d\left( x \right))$. The overall contribution of the prior distributions to our analyses (= (K+1) / N ) is very small if the number of targeted pathogen K is much smaller than the number of cases N.

The prior distribution for false positive rates is constructed from the control data, by calculating contributions of all control data points to $x\in\mathcal{X}$, using the same function $d\left( x,y \right)$. For a particular test $T$, we add the contributions from controls with positive and negative results as the Beta parameters for the false positive rate.

The smoothing parameter $h$ in Equation [5] controls the amount of local smoothing. It can be decomposed into two components: $h= \sqrt{h_{c}^{2}+h_{d}^{2}}$. Here $h_{d}$ controls the smoothness of the estimated probability density of the covariates. We use the “rule of thumb” in the density estimation literature to set $h_{d}$.^7^ For example, if N is the sample size of cases and there are two continuous covariates, then $h_{d}=N^{-1/6}$ after the covariates are scaled with mean 0.0 and variance 1.0. $h_{c}$ is used to define the neighborhood of data points. We applied knowledge of the epidemiology of the disease under evaluation to narrow the range of the parameters. For example, we choose a value 0.5 that roughly corresponds to a 3 month in enrollment period (if the total enrollment time is one year), and 12.5 years in age (if the range of the age is 50 years)

1. **Implementation details of the extended pLCM model**

Two strategies were implemented to mitigate inclusion of pathogens with characteristics that might result in unreliable model performance based on simulation experiments (see Section 2). First, we prescreened pathogen lists within each study site and each case outcome status (died vs. survived) using a stepwise procedure that excluded pathogens with very few positive TAC tests results. The second strategy flagged remaining pathogens with: a) high false positive rates among controls; b) lower odds ratio between cases and controls; and c) significant and substantial pairwise correlations of test results among controls. The effects of the covariates on flagged pathogens were further examined through stratifications and/or regression models. If the covariates did not reduce the flagged features at least locally, then flagged pathogens were considered for exclusion from the model or being combined (*Haemophilis influenzae-*all subtypes and *Haemophilis influenzae-*type B were combined).

We use non-informative priors for all parameters. The contribution of the priors was equivalent to adding one pseudo case per pathogen class into the case population. Thus, the contribution of priors was so small that our results can be considered as data driven, rather than prior distribution driven. Additionally, we set lower limits for TAC test true positive rates as 40%.

While the detection of a variety of pathogens among a large proportion of healthy controls appears to pose a challenge to our etiology study, it is the pathogens that are never detected from the healthy controls that cannot be studied without additional information to evaluate the corresponding true positive rates. In our study, influenza B virus was one such pathogen as it was rarely detected among the healthy controls.  The corresponding TAC test results cannot be used to estimate both etiology attribution and true positive rate simultaneously for influenza B, regardless of which analytic method is being used. As a result, we used average true positive rates for all other pathogens as the true positive rate of influenza B virus and deduced the etiology proportion of influenza B accordingly.

In summary, parameters in the model were estimated through the above described non-parametric Bayesian approach for both pathogen proportions and false positive rates. The model parameters at a specific covariate value were estimated locally thorough constructing local subsets of cases with more weight given to cases with covariate values closer to the local data point. Weights were calculated by a kernel function with smoothing parameters controlling the number of similar cases included in local subsets. Model convergence was assessed through trace and other diagnostic plots. Model assumptions were examined using interim model outputs by stopping the Gibbs sampler at random cycles. Model fit to input data was evaluated by comparing the fitted and observed number of positives for combinations of TAC assays. Programming and computation were conducted using the R (version 3.2.5) and SAS (version 9.3).

After a 20,000 iteration burn-in period we ran the extended pLCM for 50,000 iterations, sampling at 50 iteration intervals for a total of 1,000 samples. From the sample, posterior means and the corresponding 95% credible intervals of model parameters were then generated from the corresponding samples, either globally or stratified by covariates, or even individually for cases. Continuous two-dimensional (for age and enrollment) heat maps, as well as one-dimensional marginal or conditional curves, can also be constructed to visually reveal age and seasonal patterns of etiology proportions for individual pathogens. For each of the evaluated pathogens, the model fits the input data well with the estimated positive test results similar to the observed ones.

The computation program was written in R and can be shared upon request.

**References**

1. Wu Z, Deloria-Knoll M, Hammitt LL, Zeger SL. Partially latent class models for case-control studies of childhood pneumonia aetiology. *Journal of the Royal Statistical Society Series C: Applied Statistics.* 2016;65(1):97-114.

2. Wu Z, Deloria-Knoll M, Zeger SL. Nested partially latent class models for dependent binary data; estimating disease etiology. *Biostatistics (Oxford, England).* 2017;18(2):200-213.

3. O'Brien KL, Baggett HC, Brooks WA, et al. Introduction to the Epidemiologic Considerations, Analytic Methods, and Foundational Results From the Pneumonia Etiology Research for Child Health Study. *Clinical infectious diseases : an official publication of the Infectious Diseases Society of America.* 2017;64(suppl_3):S179-s184.

4. Deloria Knoll M, Fu W, Shi Q, et al. Bayesian Estimation of Pneumonia Etiology: Epidemiologic Considerations and Applications to the Pneumonia Etiology Research for Child Health Study. *Clinical infectious diseases : an official publication of the Infectious Diseases Society of America.* 2017;64(suppl_3):S213-s227.

5. Shang NA, ML; Liu, A; Mullany, LC; Shrag, SJ. Estimation of Pathogen Proportions of Infectious Diseases: Models, Approaches and Evaluations. *Journal of Biostatistics and Biometric Applications.* 2018;3(3).

6. Kodani M, Yang G, Conklin LM, et al. Application of TaqMan low-density arrays for simultaneous detection of multiple respiratory pathogens. *Journal of clinical microbiology.* 2011;49(6):2175-2182.

7. Silverman BW. *Density Estimation.* London: Chapman and Hall; 1986.
