# Supplementary material for: The epidemiology and estimated etiology of pathogens detected from the upper respiratory tract of adults with severe acute respiratory infections in multiple countries, 2014–2015
Source: PLoS One. 2020 Oct 19;15(10):e0240309. doi: 10.1371/journal.pone.0240309 (PMC7571682; doi:10.1371/journal.pone.0240309)
Supplement: S5 Fig — (DOCX) [file pone.0240309.s007.docx]

S5 Fig. Number of severe acute respiratory infection (SARI) patients in which RSV was detected by site and month
