# Supplementary material for: The epidemiology and estimated etiology of pathogens detected from the upper respiratory tract of adults with severe acute respiratory infections in multiple countries, 2014–2015
Source: PLoS One. 2020 Oct 19;15(10):e0240309. doi: 10.1371/journal.pone.0240309 (PMC7571682; doi:10.1371/journal.pone.0240309)
Supplement: S1 Table — (DOCX) [file pone.0240309.s008.docx]

S1 Table. Comparison of frequency of pathogens detected among severe acute respiratory infection (SARI) patients and asymptomatic adults by site

|  | **Bangladesh** | | | **China** | | | **Egypt** | | |
| --- | --- | --- | --- | --- | --- | --- | --- | --- | --- |
|  | **SARI patient** | **Asymptomatic adults** | **OR (LCL-UCL)^[[1]](#footnote-1)^** | **SARI patient** | **Asymptomatic adults** | **OR (LCL-UCL)** | **SARI patient** | **Asymptomatic adults** | **OR (LCL-UCL)** |
|  | **n (%)** | **n (%)** |  | **n (%)** | **n (%)** |  | **n (%)** | **n (%)** |  |
| Adenovirus | 5 (1.0) | 1 (0.5) | 2.0 (0.2-17.2) | 20 (3.8) | 9 (4.2) | 0.9 (0.4-2) | 2 (0.4) | 2 (1.0) | 0.4 (0.1-3.0) |
| *Bordetella pertussis* | 0 (0) | 0 (0) |  | 1 (0.2) | 1 (0.5) | 0.4 (0.03-6.5) | 1 (0.2) | 0 (0) |  |
| *Chlamydophila pneumoniae* | 1 (0.2) | 0 (0) |  | 13 (2.4) | 6 (2.8) | 0.9 (0.3-2.3) | 1 (0.2) | 0 (0) |  |
| Influenza A | 66 (13.2) | 8 (4.0) | 3.6 (1.7-7.7) | 207 (38.7) | 7 (3.2) | 18.8 (8.7-40.8) | 83 (16.5) | 0 (0) |  |
| Influenza B | 27 (5.4) | 0 (0) |  | 34 (6.4) | 0 (0) |  | 74 (14.7) | 1 (0.5) | 35.9 (45.0-259.9) |
| Influenza C | 2 (0.4) | 0 (0) |  | 2 (0.4) | 3 (1.4) | 0.3 (0.04-1.6) | 4 (0.8) | 0 (0) |  |
| Group A *Streptococcus* | 5 (1.0) | 3 (1.5) | 0.7 (0.16-2.8) | 10 (1.9) | 2 (0.9) | 2.0 (0.4-9.4) | 9 (1.8) | 5 (2.4) | 0.7 (0.3-2.2) |
| Coronavirus 229E | 3 (0.6) | 0 (0) |  | 7 (1.3) | 1 (0.5) | 2.9 (0.4-23.4) | 3 (0.6) | 1 (0.5) | 1.3 (0.1-12.2) |
| Coronavirus NL63 | 2 (0.4) | 1 (0.5) | 0.8 (0.1-8.8) | 3 (0.6) | 4 (1.9) | 0.3 (0.1-1.4) | 3 (0.6) | 1 (0.5) | 1.3 (0.1-12.1) |
| Coronavirus OC43 | 9 (1.8) | 1 (0.5) | 3.6 (0.5-28.8) | 17 (3.2) | 5 (2.3) | 1.4 (0.5-3.8) | 7 (1.4) | 4 (1.9) | 0.7 (0.2-2.5) |
| Coronavirus HKU1 | 3 (0.6) | 0 (0) |  | 2 (0.4) | 3 (1.4) | 0.3 (0.04-1.6) | 1(0.2) | 1 (0.5) | 0.4 (0.03-6.7) |
| *Haemophilus influenzae* | 128 (25.7) | 44 (22.2) | 1.2 (0.8-1.8) | 92 (17.2) | 46 (21.3) | 0.8 (0.5-1.1) | 91 (18.1) | 62 (29.7) | 0.5 (0.4-0.8) |
| Human metapneumovirus | 1 (0.2) | 1 (0.5) | 0.4 (0.02-6.4) | 15 (2.8) | 2 (0.9) | 3.1 (0.7-13.6) | 10 (2.0) | 1 (0.5) | 4.2 (0.5-33.2) |
| *Klebsiella* *pneumoniae* | 67 (13.4) | 15 (7.6) | 1.9 (1.1-3.4) | 19 (3.6) | 9 (4.2) | 0.9 (0.4-1.9) | 30 (6.0) | 9 (4.3) | 1.4 (0.7-3.0) |
| *Legionella* species | 0 (0) | 0 (0) |  | 1 (0.2) | 0 (0) |  | 0 (0) | 0 (0) |  |
| *Moraxella catarrhalis* | 84 (16.8) | 36 (18.2) | 0.9 (0.6-1.4) | 27 (5.1) | 13 (6.0) | 0.8 (0.4-1.6) | 50 (9.9) | 48 (23.0) | 0.4 (0.2-0.6) |
| *Mycoplasma pneumoniae* | 1 (0.2) | 1 (0.5) | 0.4 (0.02-6.4) | 10 (1.9) | 1 (0.5) | 4.1 (0.5-32.3) | 2 (0.4) | 0 (0) |  |
| *Mycobacterium tuberculosis* | 3 (0.6) | 0 (0) |  | 0 (0) | 0 (0) |  | 0 (0) | 0 (0) |  |
| Human parainfluenza virus 1 | 5 (1.0) | 0(0) |  | 2 (0.4) | 0(0) |  | 0 (0) | 0 (0) |  |
| Human parainfluenza virus 2 | 0 (0) | 0(0) |  | 5 (0.9) | 1(0.5) | 2.0 (0.2-17.5) | 0 (0) | 0 (0) |  |
| Human parainfluenza virus 3 | 12 (2.4) | 1 (0.5) | 4.9 (0.6-37.6) | 3 (0.6) | 2 (0.9) | 0.6 (0.1-3.6) | 9 (1.8) | 0 (0) |  |
| Human parainfluenza virus 4 | 6 (1.2) | 0 (0) |  | 0 (0) | 1 (0.5) |  | 1 (0.2) | 0 (0) |  |
| *Pneumocystis jiroveci* (PCP) | 0 (0) | 0 (0) |  | 1 (0.2) | 0 (0) |  | 1 (0.2) | 0 (0) |  |
| *Pseudomonas aeruginosa* | 24 (4.8) | 4 (2.0) | 2.5 (0.8-7.2) | 8 (1.5) | 1 (0.5) | 3.3 (0.4-26.3) | 0 (0) | 0 (0) |  |
| Respiratory syncytial virus^[[2]](#footnote-2)^ | 17 (3.4) | 2 (1.0) | 3.5 (0.8-15.1) | NA | NA | NA | 8 (1.6) | 0 (0) |  |
| *Staphylococcus aureus* | 62 (12.4) | 22 (11.1) | 1.1 (0.7-1.9) | 19 (3.6) | 7 (3.2) | 1.1 (0.5-2.7) | 44 (8.8) | 25 (12.0) | 0.7 (0.4-1.2) |
| *Streptococcus pneumoniae* | 165 (33.1) | 57 (28.8) | 1.2 (0.9-1.8) | 59 (11.1) | 16 (7.4) | 1.6 (0.9-2.8) | 65 (12.9) | 61 (29.2) | 0.4 (0.2-0.5) |
| Rhinovirus/Enterovirus | 82 (16.4) | 13 (6.6) | 2.8 (1.5-5.2) | 71 (14.0) | 23 (10.9) | 1.3 (0.8-2.2) | 47 (9.5) | 18 (8.8) | 1.1 (0.6-1.9) |

Table S1 Continued: Comparison of frequency of pathogens detected among severe acute respiratory infection (SARI) patients and asymptomatic adults by site

|  | **Guatemala** | | | **Kenya** | | | **Thailand** | | |
| --- | --- | --- | --- | --- | --- | --- | --- | --- | --- |
|  | **SARI Patient** | **Asymptomatic adults** | **OR (LCL-UCL)** | **SARI Patient** | **Asymptomatic adults** | **OR (LCL-UCL)** | **SARI Patient** | **Asymptomatic adults** | **OR (LCL-UCL)** |
|  | **n (%)** | **n (%)** |  | **n (%)** | **n (%)** |  | **n (%)** | **n (%)** |  |
| Adenovirus | 3 (1.0) | 0 (0) |  | 3(1.61) | 5 (4.13) | 0.4 (0.1-1.6) | 1 (0.3) | 5 (2.3) | 0.1 (0.01-1.0) |
| *Bordetella pertussis* | 0 (0) | 1 (0.6) |  | 0(0) | 0 (0) |  | 1 (0.3) | 1 (0.5) | 0.6 (0.04-9.8) |
| *Chlamydophila pneumoniae* | 2 (0.7) | 0 (0) |  | 0(0) | 0 (0) |  | 1 (0.3) | 1 (0.5) | 0.6 (0.04-9.8) |
| Influenza A | 30 (9.9) | 1 (0.6) | 19.1 (2.6-141.2) | 20(10.75) | 1 (0.8) | 14.5 (1.9-109.2) | 37 (10.4) | 0 (0) |  |
| Influenza B | 5 (1.7) | 1 (0.6) | 2.9 (0.3-25.1) | 0(0) | 0 (0) |  | 44 (12.3) | 1 (0.5) | 30.4 (4.2-222.1) |
| Influenza C | 0 (0) | 0 (0) |  | 0(0) | 0 (0) |  | 0 (0) | 0 (0) |  |
| Group A *Streptococcus* | 9 (3.0) | 6 (3.5) | 0.9 (0.3-2.5) | 2(1.08) | 0 (0) |  | 4 (1.1) | 2 (0.9) | 1.2 (0.2-6.7) |
| Coronavirus 229E | 4 (1.3) | 0 (0) |  | 0(0) | 0 (0) |  | 1 (0.3) | 1 (0.5) | 0.6 (0.04-9.8) |
| Coronavirus NL63 | 0 (0) | 0 (0) |  | 3(1.61) | 1 (0.8) | 2.0 (0.2-19.1) | 2 (0.6) | 0 (0) |  |
| Coronavirus OC43 | 2 (0.7) | 0 (0) |  | 5(2.69) | 2 (1.7) | 1.6 (0.3-8.3) | 0 (0) | 0 (0) |  |
| Coronavirus HKU1 | 3 (1.0) | 2 (1.2) | 0.9 (0.1-5.2) | 0(0) | 0 (0) |  | 2 (0.6) | 1 (0.5) | 1.2 (0.1-13.5) |
| *Haemophilus influenzae* | 60 (19.9) | 49 (28.2) | 0.6 (0.4-0.99) | 36(19.35) | 20 (16.5) | 1.2 (0.7-2.2) | 133 (37.3) | 77 (35.5) | 1.1 (0.8-1.5) |
| Human metapneumovirus | 9 (2.98) | 0 (0) |  | 4(2.15) | 0 (0) |  | 2 (0.6) | 0 (0) |  |
| *Klebsiella* *pneumoniae* | 17 (5.6) | 18 (10.3) | 0.5 (0.3-1.0) | 16(8.84) | 6 (5.0) | 1.9 (0.7-4.9) | 71 (19.9) | 48 (22.1) | 0.9 (0.6-1.3) |
| *Legionella* species | 0(0) | 1 (0.6) |  | 0(0) | 0 (0) |  | 1 (0.3) | 0 (0) |  |
| *Moraxella catarrhalis* | 38 (12.6) | 19 (10.9) | 1.2 (0.7-2.1) | 25(13.44) | 12 (9.9) | 1.4 (0.7-2.9) | 45 (12.6) | 13 (6.0) | 2.3 (1.2-4.3) |
| *Mycoplasma pneumoniae* | 4 (1.3) | 0 (0) |  | 0(0) | 0 (0) |  | 2 (0.6) | 0 (0) |  |
| *Mycobacterium tuberculosis* | 1 (0.4) | 0 (0) |  | 0(0) | 0 (0) |  | 1 (0.3) | 0 (0) |  |
| Human parainfluenza virus 1 | 1 (0.3) | 1 (0.6) | 0.6 (0.04-9.3) | 3(1.61) | 0 (0) |  | 5 (1.4) | 1 (0.5) | 3.1 (0.4-26.4) |
| Human parainfluenza virus 2 | 1 (0.3) | 0 (0) |  | 2(1.08) | 0 (0) |  | 3 (0.8) | 0 (0) |  |
| Human parainfluenza virus 3 | 8 (2.7) | 0 (0) |  | 1(0.54) | 0 (0) |  | 10 (2.8) | 0 (0) |  |
| Human parainfluenza virus 4 | 3 (1.0) | 0 (0) |  | 1(0.56) | 0 (0) |  | 2 (0.6) | 0 (0) |  |
| *Pneumocystis jiroveci* (PCP) | 2 (0.7) | 0 (0) |  | 2(1.08) | 0 (0) |  | 4 (1.1) | 0 (0) |  |
| *Pseudomonas aeruginosa* | 13 (4.3) | 1 (0.6) | 7.8 (1.0-60.0) | 10(5.56) | 10 (8.3) | 0.7 (0.3-1.6) | 19 (5.3) | 15 (6.9) | 0.8 (0.4-1.5) |
| Respiratory syncytial virus | 13 (4.3) | 0 (0) |  | 3(1.61) | 0 (0) |  | 32 (9.0) | 2 (0.9) | 10.6 (2.5-44.6) |
| *Staphylococcus aureus* | 36 (11.9) | 24 (13.8) | 0.9 (0.5-1.5) | 37(19.89) | 17 (14.1) | 1.5 (0.8-2.8) | 27 (7.6) | 25 (11.5) | 0.6 (0.4-1.1) |
| *Streptococcus pneumoniae* | 75 (24.8) | 45 (25.9) | 1.0 (0.6-1.5) | 86(46.49) | 50 (41.3) | 1.2 (0.8-2.0) | 114 (31.9) | 57 (26.7) | 1.3 (0.9-1.9) |
| Rhinovirus/Enterovirus | 69 (27.1) | 4 (2.3) | 15.8 (5.6-44.1) | 31(19.14) | 11 (9.9) | 2.2 (1.0-4.5) | 38 (10.6) | 5 (2.3) | 5.1 (2.0-13.0) |

1. OR=Odds ratio; LCL=Lower confidence limit; UCL=Upper confidence limit [↑](#footnote-ref-1)
2. RSV assay results were not included from China due to contamination [↑](#footnote-ref-2)
