# Supplementary material for: The epidemiology and estimated etiology of pathogens detected from the upper respiratory tract of adults with severe acute respiratory infections in multiple countries, 2014–2015
Source: PLoS One. 2020 Oct 19;15(10):e0240309. doi: 10.1371/journal.pone.0240309 (PMC7571682; doi:10.1371/journal.pone.0240309)
Supplement: S2 Table — (DOCX) [file pone.0240309.s009.docx]

S2 Table. Estimated attributed etiology of severe acute respiratory infection (SARI) patients by pathogen and site

|  | **Attributed etiology**  **Mean, Percent of SARI episodes (Lower credible interval-Upper credible interval)** | | | | | |
| --- | --- | --- | --- | --- | --- | --- |
|  | Bangladesh | China^[[1]](#footnote-1)^ | Egypt | Guatemala | Kenya | Thailand |
| **Adenovirus** | 0.8 (0.2-2.0) | 1.4 (0.2-3.8) |  | 1.3 (0.8-2.8) |  |  |
| ***C. pneumoniae*** |  | 0.3 (0.1-0.7) |  |  |  |  |
| **Coronavirus 229E (HCV1)** | 0.5 (0.3-1.1) | 0.7 (0.3-1.7) | 0.2 (0.1-0.5) | 0.6 (0.4-1.7) |  |  |
| **Coronavirus OC43 (HCV3)** | 1.6 (0.7-3.1) | 0.6 (0.1-1.5) | 0.3 (0.1-0.7) |  | 1.4 (0.3-4.3) |  |
| **Group A *Streptococcus*** | 0.4 (0.2-1.2) | 1.4 (0.2-3.2) | 1.9 (0.8-3.9) | 1.8 (0.7-4.0) |  | 0.8 (0.5-1.4) |
| ***H. influenzae*- all types** | 2.7 (0.2-6.8) | 1.0 (0.2-2.4) | 0.4 (0.1-1.5) | 0.5 (0.1-1.3) | 2.2 (0.2-7.6) | 1.4 (0.2-5.7) |
| **Human metapneumovirus** |  | 4.2 (2.2-7.2) | 2.5 (1.3-4.6) | 4.7 (2.6-8.3) | 3.9 (2.4-8.1) |  |
| **Influenza A** | 15.0 (10.0-20.6) | 54.4 (37.7-66.7) | 23.3 (16.5-29.3) | 12.1 (8.2-17.1) | 15.2 (10.3-21.4) | 14.5 (10.2-18.4) |
| **Influenza B** | 7.0 (6.5-7.8) | 8.2 (7.5-9.0) | 19.1 (17.5-21.0) | 1.9 (1.8-2.1) |  | 17.2 (15.7-18.9) |
| **Influenza C** |  |  | 1.1 (0.6-2.3) |  |  |  |
| ***K. pneumoniae*** | 5.2 (1.2-10) | 0.3 (0.1-1.0) | 1.1 (0.1-3.5) | 0.4 (0.2-1.2) | 5.9 3.0-11.2) | 0.7 (0.1-2.8) |
| ***M. catarrhalis*** | 0.7 (0.1-2.2) | 1.1 (0.1-2.5) | 0.3 (0.1-1.0) | 1.6 (0.4-3.2) | 2.6 (0.4-5.6) | 6.1 (3.3-10.2) |
| ***M. pneumoniae*** |  | 2.3 (1.4-4.3) |  |  |  |  |
| ***P. aeruginosa*** | 4.8 (2.7-8.6) | 1.3 (0.4-2.7) |  | 3.7 (2.2-6.9) | 1.0 (0.4-2.8) | 0.8 (0.2-2.3) |
| **Parainfluenza virus 1** | 1.1 (0.8-2.1) |  |  |  | 2.5 (1.8-5.2) | 1.7 (1.0-3.6) |
| **Parainfluenza virus 3** | 2.1 (1.3-3.6) |  | 2.7 (1.8-5.1) | 4.4 (3.0-8.4) |  | 3.4 (2.6-6.0) |
| **Parainfluenza virus 4** | 2.3 (1.2-4.2) |  |  | 1.4 (0.6-3.3) |  |  |
| **Respiratory syncytial virus** | 3.9 (1.5-7.4) |  | 1.8(0.9-3.4) | 6.4 (3.6-10.6) | 3.6 (2.0-7.3) | 14.6 (7.9-23.6) |
| ***S. aureus*** | 2.4 (0.2-7.2) | 2.1 (0.4-4.5) | 0.7 (0.2-2.7) | 1.4 (0.2-4.6) | 5.7 (0.4-17.61) | 1.5 (0.2-5.2) |
| ***S. pneumoniae*** | 1.9 (0.1-7.0) | 2.6 (1.2-4.6) | 0.3 (0.1-0.7) | 4.3 (0.2-8.8) | 2.1 (0.4-8.1) | 1.8 (0.2-6.4) |
| **Rhinovirus/enterovirus** | 15.6 (8.8-22.7) | 11.5 (5.4-18.1) | 1.8 (0.1-5.7) | 42.6 (24.9-56.3) | 14.1 (2.8-27.5) | 13.8 (8.3-19.3) |
| **Others^[[2]](#footnote-2)^** | 32.0 (19.6-45.1) | 6.8 (5.6-27.2) | 42.7 (34.0-52.1) | 10.9 (0.9-30.2) | 39.8 (21.0-57.2) | 22.0 (8.4-34.8) |

1. RSV was excluded from modeling in China due to contamination [↑](#footnote-ref-1)
2. Includes all other pathogens that could cause severe acute respiratory illness [↑](#footnote-ref-2)
