# Supplementary material for: The epidemiology and estimated etiology of pathogens detected from the upper respiratory tract of adults with severe acute respiratory infections in multiple countries, 2014–2015
Source: PLoS One. 2020 Oct 19;15(10):e0240309. doi: 10.1371/journal.pone.0240309 (PMC7571682; doi:10.1371/journal.pone.0240309)
Supplement: S3 Table — (DOCX) [file pone.0240309.s010.docx]

**S3 Table.** True positive rate* of the TAC assays

|  | **Mean** | **Lower confidence limit** | **Upper confidence limit** |
| --- | --- | --- | --- |
| **Bacteria** |  |  |  |
| *C. pneumoniae* | 73.6 | 41.4 | 99.9 |
| Group A *Streptococcus* | 81.7 | 43.8 | 100.0 |
| *H. influenzae*- all types | 91.2 | 72.8 | 100.0 |
| *K. pneumoniae* | 81.0 | 51.5 | 99.9 |
| *M. catarrhalis* | 86.4 | 55.6 | 100.0 |
| *M. pneumoniae* | 74.7 | 41.8 | 99.9 |
| *P. aeruginosa* | 79.0 | 44.9 | 99.9 |
| *S. aureus* | 71.2 | 43.5 | 99.5 |
| *S. pneumoniae* | 87.9 | 69.9 | 100.0 |
| ***Viruses*** |  |  |  |
| Adenovirus | 70.3 | 41.4 | 99.7 |
| Coronavirus 229E | 84.7 | 46.6 | 100.0 |
| Coronavirus OC43 | 81.7 | 45.9 | 100.0 |
| Human metapneumovirus | 63.3 | 40.7 | 99.6 |
| Influenza A | 69.3 | 55.4 | 93.0 |
| Influenza C | 75.2 | 41.4 | 100.0 |
| Respiratory syncytial virus | 64.1 | 40.5 | 99.8 |
| Rhinovirus/enterovirus | 64.0 | 44.0 | 99.4 |
| Parainfluenza virus 1 | 79.0 | 42.2 | 100.0 |
| Parainfluenza virus 3 | 78.6 | 43.4 | 99.9 |
| Parainfluenza virus 4 | 67.1 | 41.0 | 99.7 |

*For a given pathogen that is determined to be the etiology of SARI, the proportion of SARI cases with a positive test for that pathogen
