# Supplementary material for: The epidemiology and estimated etiology of pathogens detected from the upper respiratory tract of adults with severe acute respiratory infections in multiple countries, 2014–2015
Source: PLoS One. 2020 Oct 19;15(10):e0240309. doi: 10.1371/journal.pone.0240309 (PMC7571682; doi:10.1371/journal.pone.0240309)
Supplement: S4 Table — (DOCX) [file pone.0240309.s011.docx]

**S4 Table.** Percentage of severe acute respiratory infection (SARI) patients and asymptomatic adults in which pathogens were detected

|  | **Bangladesh** | | **China** | | **Egypt** | | **Guatemala** | | **Kenya** | | **Thailand** | | **Total** | |
| --- | --- | --- | --- | --- | --- | --- | --- | --- | --- | --- | --- | --- | --- | --- |
|  | **SARI patient (499) %** | **Asymptomatic adults**  **(198) %** | **SARI patient (539) %** | **Asymptomatic adults**  **(216) %** | **SARI patient (504) %** | **Asymptomatic adults**  **(209) %** | **SARI patient (304) %** | **Asymptomatic adults**  **(174) %** | **SARI patient (187) %** | **Asymptomatic adults**  **(121)** | **%SARI patient (357) %** | **Asymptomatic adults**  **(217) %** | **SARI patient (2388) %** | **Asymptomatic adults**  **(1135) %** |
| Adenovirus | 1.0 | 0.5 | 3.7 | 4.2 | 0.4 | 1.0 | 1.0 | 0.0 | 1.6 | 4.1 | 0.3 | 2.3 | 1.4 | 1.9 |
| B. pertussis | 0.0 | 0.0 | 0.2 | 0.5 | 0.2 | 0.0 | 0.0 | 0.6 | 0.0 | 0.0 | 0.3 | 0.5 | 0.1 | 0.3 |
| C. pneumoniae | 0.2 | 0.0 | 2.4 | 2.8 | 0.2 | 0.0 | 0.7 | 0.0 | 0.5 | 0.0 | 0.3 | 0.5 | 0.8 | 0.6 |
| Coronavirus 229E (HCV1) | 0.6 | 0.0 | 1.3 | 0.5 | 0.6 | 0.5 | 1.3 | 0.0 | 0.0 | 0.0 | 0.3 | 0.5 | 0.8 | 0.3 |
| Coronavirus HKU1 (HVV4) | 0.6 | 0.0 | 0.4 | 1.4 | 0.2 | 0.5 | 1.0 | 1.1 | 0.0 | 0.0 | 0.6 | 0.5 | 0.5 | 0.6 |
| Coronavirus NL63 (HCV2) | 0.4 | 0.5 | 0.6 | 1.9 | 0.6 | 0.5 | 0.0 | 0.0 | 1.6 | 0.8 | 0.6 | 0.0 | 0.5 | 0.6 |
| Coronavirus OC43 (HCV3) | 1.8 | 0.5 | 3.2 | 2.3 | 1.4 | 1.9 | 0.7 | 0.0 | 2.7 | 1.7 | 0.0 | 0.0 | 1.7 | 1.1 |
| Group A Strep | 1.0 | 1.5 | 1.9 | 0.9 | 1.8 | 2.4 | 3.0 | 3.4 | 1.1 | 0.0 | 1.1 | 0.9 | 1.6 | 1.6 |
| H. influenzae- all types | 25.7 | 22.2 | 17.1 | 21.3 | 18.1 | 29.7 | 19.9 | 28.2 | 19.3 | 16.5 | 37.3 | 35.5 | 22.6 | 26.3 |
| Human metapneumovirus | 0.2 | 0.5 | 2.8 | 0.9 | 2.0 | 0.5 | 3.0 | 0.0 | 2.1 | 0.0 | 0.6 | 0.0 | 1.7 | 0.4 |
| Influenza A | 13.2 | 4.0 | 38.4 | 3.2 | 16.5 | 0.0 | 9.9 | 0.6 | 10.7 | 0.8 | 10.4 | 0.0 | 18.6 | 1.5 |
| Influenza B | 5.4 | 0.0 | 6.3 | 0.0 | 14.7 | 0.5 | 1.7 | 0.6 | 0.0 | 0.0 | 12.3 | 0.5 | 7.7 | 0.3 |
| Influenza C | 0.4 | 0.0 | 0.4 | 1.4 | 0.8 | 0.0 | 0.0 | 0.0 | 0.0 | 0.0 | 0.0 | 0.0 | 0.3 | 0.3 |
| K. pneumoniae | 13.4 | 7.6 | 3.5 | 4.2 | 6.0 | 4.3 | 5.6 | 10.3 | 8.6 | 5.0 | 19.9 | 22.1 | 9.2 | 9.3 |
| Legionella | 0.0 | 0.0 | 0.2 | 0.0 | 0.0 | 0.0 | 0.0 | 0.6 | 0.0 | 0.0 | 0.3 | 0.0 | 0.1 | 0.1 |
| M. catarrhalis | 16.8 | 18.2 | 5.0 | 6.0 | 9.9 | 23.0 | 12.6 | 10.9 | 13.4 | 9.9 | 12.6 | 6.0 | 11.3 | 12.4 |
| M. pneumoniae | 0.2 | 0.5 | 1.9 | 0.5 | 0.4 | 0.0 | 1.3 | 0.0 | 0.0 | 0.0 | 0.6 | 0.0 | 0.8 | 0.2 |
| M. tuberculosis | 0.6 | 0.0 | 0.0 | 0.0 | 0.0 | 0.0 | 0.4 | 0.0 | 0.0 | 0.0 | 0.3 | 0.0 | 0.2 | 0.0 |
| P. aeruginosa | 4.8 | 2.0 | 1.5 | 0.5 | 1.2 | 0.5 | 4.3 | 0.6 | 5.3 | 8.3 | 5.3 | 6.9 | 3.4 | 2.8 |
| P. jiroveci | 0.0 | 0.0 | 0.2 | 0.0 | 0.2 | 0.0 | 0.7 | 0.0 | 1.1 | 0.0 | 1.1 | 0.0 | 0.4 | 0.0 |
| Parainfluenza virus 1 | 1.0 | 0.0 | 0.4 | 0.0 | 0.0 | 0.0 | 0.3 | 0.6 | 1.6 | 0.0 | 1.4 | 0.5 | 0.7 | 0.2 |
| Parainfluenza virus 2 | 0.0 | 0.0 | 0.9 | 0.5 | 0.0 | 0.0 | 0.3 | 0.0 | 1.1 | 0.0 | 0.8 | 0.0 | 0.5 | 0.1 |
| Parainfluenza virus 3 | 2.4 | 0.5 | 0.6 | 0.9 | 1.8 | 0.0 | 2.6 | 0.0 | 0.5 | 0.0 | 2.8 | 0.0 | 1.8 | 0.3 |
| Parainfluenza virus 4 | 1.2 | 0.0 | 0.0 | 0.5 | 0.2 | 0.0 | 1.0 | 0.0 | 0.5 | 0.0 | 0.6 | 0.0 | 0.5 | 0.1 |
| Respiratory Syncytial Virus | 3.4 | 1.0 |  |  | 1.6 | 0.0 | 4.3 | 0.0 | 1.6 | 0.0 | 9.0 | 0.9 | 3.9 | 0.4 |
| S. aureus | 12.4 | 11.1 | 3.5 | 3.2 | 8.7 | 12.0 | 11.9 | 13.8 | 19.8 | 14.0 | 7.6 | 11.5 | 9.4 | 10.6 |
| S. pneumoniae | 33.1 | 28.8 | 10.9 | 7.4 | 12.9 | 29.2 | 24.8 | 25.9 | 46.0 | 41.3 | 31.9 | 26.3 | 23.6 | 25.2 |
| Rhinovirus/Enterovirus | 18.4 | 7.1 | 14.1 | 11.1 | 9.7 | 9.1 | 24.2 | 2.9 | 18.7 | 9.9 | 12.0 | 2.3 | 15.4 | 7.0 |
| Parainfluenza viruses | 4.6 | 0.5 | 1.9 | 1.9 | 2.0 | 0.0 | 4.3 | 0.6 | 3.7 | 0.0 | 5.6 | 0.5 | 3.5 | 0.6 |
| Coronaviruses | 3.4 | 1.0 | 5.4 | 6.0 | 2.8 | 3.3 | 3.0 | 1.1 | 4.3 | 2.5 | 1.4 | 0.9 | 3.4 | 2.6 |
